# Fully First-Principles Surface Spectroscopy with Machine Learning

**DOI:** 10.1021/acs.jpclett.3c01989

**Published:** 2023-09-06

**Authors:** Yair Litman, Jinggang Lan, Yuki Nagata, David M. Wilkins

**Affiliations:** † Yusuf Hamied Department of Chemistry, University of Cambridge, Lensfield Road, Cambridge CB2 1EW, U.K.; ‡ Max Planck Institute for Polymer Research, Ackermannweg 10, 55128 Mainz, Germany; ¶ Department of Chemistry, New York University, New York, New York 10003, United States; § Simons Center for Computational Physical Chemistry at New York University, New York, New York 10003, United States; ∥ Centre for Quantum Materials and Technologies School of Mathematics and Physics, Queen’s University Belfast, Belfast BT7 1NN, Northern Ireland, United Kingdom

## Abstract

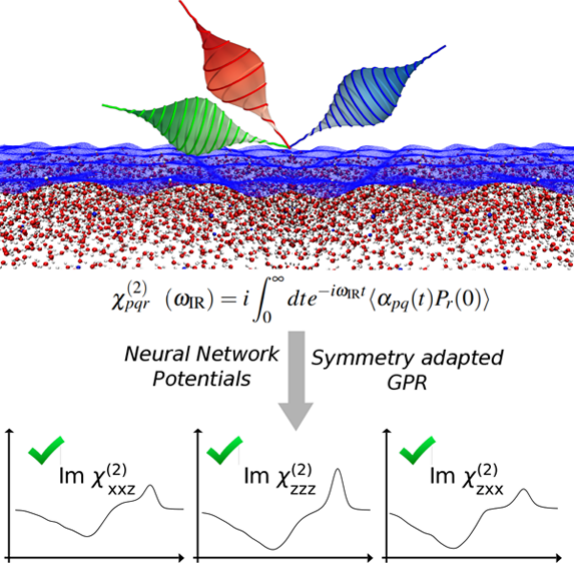

Our current understanding of the structure and dynamics of aqueous interfaces at the molecular level has grown substantially due to the continuous development of surface-specific spectroscopies, such as vibrational sum-frequency generation (VSFG). As in other vibrational spectroscopies, we must turn to atomistic simulations to extract all of the information encoded in the VSFG spectra. The high computational cost associated with existing methods means that they have limitations in representing systems with complex electronic structure or in achieving statistical convergence. In this work, we combine high-dimensional neural network interatomic potentials and symmetry-adapted Gaussian process regression to overcome these constraints. We show that it is possible to model VSFG signals with fully *ab initio* accuracy using machine learning and illustrate the versatility of our approach on the water/air interface. Our strategy allows us to identify the main sources of theoretical inaccuracy and establish a clear pathway toward the modeling of surface-sensitive spectroscopy of complex interfaces.

Soft matter interfaces, including aqueous interfaces, solid/liquid interfaces, and liquid/liquid interfaces, are ubiquitous in nature and play a crucial role in many important processes, such as (electro)catalytic/electrochemical applications,^1^ atmospheric aerosol–gas exchanges,^2^ and mineral dissolution.^3^ These processes are governed by the molecular level interaction between the molecules at the interface and the other (electrified) molecules/materials.

To probe the interfacial response, a technique must isolate the signal of the relatively few surface molecules at the surfaces from the enormous contribution due to the bulk.^4^ Vibrational sum frequency generation (VSFG) is a technique where IR and visible beams are spatially and temporally overlapped and the signal generated at the sum of the input beam frequencies is measured. VSFG is a second-order nonlinear optical process, and as in any other even-order nonlinear optical techniques, the centro-symmetric bulk contributions vanish due to the symmetry of the second-order susceptibility, χ^(2)^, making their signal surface-specific.^5^ The VSFG signal further possesses molecular specificity: a VSFG signal is enhanced when the IR frequency is resonant with an interfacial molecular vibration. When combining probes with different polarizations, VSFG can provide information on the orientation of interfacial molecules,^6−8^ the depth profile of the interfacial molecules,^9^ and molecular chirality.^10^ This makes VSFG a powerful method to characterize the identity, structure, and interaction of the molecules at interfaces.

Experimental VSFG data alone are normally insufficient to connect spectroscopic observables with molecular structure, and atomistic simulations are required to achieve a microscopic understanding. The theoretical calculation of VSFG spectra is more challenging than that of more traditional spectroscopies such as linear IR and Raman, since relatively long simulation times (on the order of nanoseconds) are required to converge the statistics and guarantee that the signal in the bulk-like (centrosymmetric) regions vanishes.^11,12^

The vibrational resonant component of the second-order susceptibility, χ_*pqr*_^(2)^, in an electronically nonresonant condition, can be computed as^11^
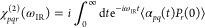
1where α_*pq*_ is the *pq* component of the polarizability tensor, ω_IR_ is the frequency of the IR pulse, and *P*_*r*_ is the *r* component of the polarization vector. The evaluation of eq 1 requires an accurate representation of three objects, namely, (i) potential energy surface (PES), (ii) polarization surface (*P*-S), and (iii) polarizability surface (α-S).

Several approximations have been applied to calculate the VSFG spectrum of aqueous systems with varying degrees of success. The first group of studies use empirical polarizable models of the dipole moment and polarizability based on *ab initio* data.^11−13^ The drawback of such strategies is that accurate polarizable models are normally burdensome to construct and validate and cannot handle bond breaking or formation. The second group uses the projection of the transition dipole and polarizability moments into atomic velocities, leading to the surface-specific velocity–velocity correlation function formalism (ssVVCF) approach.^14,15^ This approximation cannot predict the spectral differences observed with different combinations of laser polarization, since due to the approximations involved, it (incorrectly) predicts χ_xxz_^(2)^ = χ_zzz_^(2)^ and χ_zxz_^(2)^ = 0. Furthermore, it is unable to capture important spectral features originating from vibrational coupling.^16−18^

In this Letter, to overcome the problems inherent in “modeling the dipole moment and polarizability, the slow convergence of the time correlation function, and the accurate prediction of the VSFG spectra at different polarization combinations, we propose the combination of several machine-learning (ML) methods. The PES is evaluated using either density functional theory (DFT) or Behler–Parrinello high-dimensional neural networks (HDNNPs),^19^ and *P*-S and α-S are evaluated by a symmetry-adapted Gaussian process regression (SA-GPR) scheme that enables the prediction of tensorial quantities of arbitrary order.^20,21^ The proposed scheme reduces the calculation cost of the fully first-principles modeling by more than 3 orders of magnitude, enabling the simulation of VSFG spectra at various interfaces at any polarization combination.

The modeling of aqueous solution/air interfaces is normally performed by simulating the system under study in a slab geometry, in which the system extends infinitely along two dimensions and has finite size along the third dimension where it is sandwiched by regions of vacuum. This geometry gives rise to two interfaces that generate VSFG signals with opposite signs that cancel each other and lead to a vanishing signal. Thus, it is not possible to obtain any meaningful spectra if one uses *P* and α of a total slab geometry in eq 1. As we discuss below, our ML approach offers an elegant and data-driven solution to this issue.

We start by describing the training of the *P*-S and α-S using SA-GPR. The reference data were obtained using DFT at the PBE and PBE0 level and POLY2VS, a polarizable water force field developed by Tanimura and co-workers.^22^ The PBE and PBE0 data sets are obtained from first-principles calculations and constitute the ultimate target of this work. The POLY2VS data set provides access to a molecular decomposition of the α and *P* quantities, allowing us to critically assess the performance of the ML models. Moreover, we considered two types of data sets, one made up exclusively of bulk structures and a second one made of water clusters from monomers up to hexamers (see a more detailed description of the data set and the training procedure in the Supporting Information).

In Table 1, we summarize the different models for the dielectric properties considered in this work. To enable comparisons between data sets, the error estimates are computed as the root-mean-square error (RMSE) percentage of the intrinsic deviation of the data set and expressed per water molecule. The SA-GPR models can accurately learn *P* and α with errors below a few percentages of intrinsic variation in the training set. These values represent an error below 8.0 × 10^–3^ D/atom and 1 × 10^–3^ Å^3^/atom for |*P*| and Tr[α], respectively. In the Supporting Information, we report the correlation plots and learning curves of the ML models, together with a brief analysis of the training errors.

**Table 1:** Root Mean Squared Error (RMSE) for SA-GPR Models of Polarization per Atom (Debye) and Polarizability Per Atom (Å^3^)^a^

Model Name	Reference	Configurations	Polarization (*P*)	Polarizability (α)
ML-POLY-A	POLY2VS	Bulk Water	7.6 × 10^–4^ (1.7%)	2.7 × 10^–3^ (13%)
ML-POLY-B	POLY2VS	Water Clusters	5.4 × 10^–4^ (0.5%)	2.2 × 10^–3^ (3%)
ML-PBE-A	DFT (PBE)	Bulk Water	1.0 × 10^–3^ (0.8%)	2.6 × 10^–3^ (13%)
ML-PBE0-A	DFT (PBE0)	Bulk Water	2.2 × 10^–3^ (0.8%)	–

a Numbers in parentheses give the RMSE as a percentage of the intrinsic deviation in the training set.

Global accuracy estimators, such as the RMSE and absolute error presented above, are useful indicators of the overall performance of the ML models. However, when the learned quantities are used for further calculations, as we do here for χ^(2)^, it is normally not possible to translate RMSE values directly into an objective accuracy measure of the final target. This is mainly because of the difficulty in performing a rigorous error propagation of the uncertainties in eq 1 but also due to the lack of a unique set of descriptors to quantify how “good” a predicted spectrum is. In this work, we take what we consider to be the strictest validation test and use VSFG spectra to judge the accuracy of our models.

The calculation of χ^(2)^ for a slab geometry needs to be done with care since the contributions of opposite interfaces interfere destructively and lead to a vanishing response. Assuming that the slab thickness is large enough to accommodate a bulk region in the middle and that a molecular decomposition of *P* is available, it is standard practice to set to zero or flip the sign of the molecular dipoles below the center of mass of the slab to avoid this cancellation.^14,15^ However, molecular dipoles are not observables, and therefore, they are arbitrary in nature. In *ab initio* calculations the molecular decomposition can be achieved by using maximally localized Wannier functions,^23,24^ which incurs the difficulty of requiring direct *ab initio* simulations.^25^ Here, we instead utilized an unbiased data-driven approach. Since we are using an atom-centered decomposition to represent the atomic environments, the SA-GPR predictions can be expressed as a sum of molecular contributions as
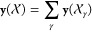
2where γ represents the set of indices that corresponds to a given molecule, and  and  the atomic environment of the trial configuration and the atomic environment of the γ water molecule, respectively. In this way, SA-GPR can be applied to the calculation of VSFG using eq 1 for slab geometries by applying the following modification to eq 2 for the *P* predictions
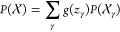
3where the surface plane is assumed to be parallel to the *xy* plane, *z*_γ_ is the *z*-coordinate of the γ molecule, and
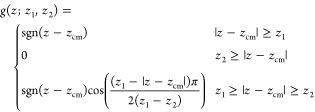
4where *z*_cm_ is the z-coordinate of the slab center of mass and *z*_1_ and *z*_2_ are two parameters that define the transition between the interfacial and bulk regions. The first condition in eq 4 flips the sign of the molecular contributions to the polarization that emerge from water molecules that are in the vicinity of the bottom interface, the second condition sets to zero the contributions to the polarization corresponding to water molecules that are in the vicinity of the slab center, and the third condition is a smooth transition between the previous two conditions. In this way, the signal of the opposite interfaces interfere constructively, leading to a nonvanishing VSFG signal.

Irrespective of the polarization, the Imχ^(2)^ spectrum of the water/air interface presents a sharp positive peak centered at 3700 cm^–1^ and a broad band in the 3200–3500 cm^–1^ region with a negative amplitude. The peak is due to the dangling (free) O–H group of the topmost interfacial water layer, and the band is associated with O–H groups of the interfacial water which form hydrogen bonds with other water molecules. The sign of the amplitudes in the Imχ^(2)^ spectrum can be directly associated with the orientation of the water molecules. More specifically, positive (negative) amplitudes imply that the O–H group points toward the air (toward the bulk) phase. χ_pqr_^(2)^ has only 3 independent elements: χ_zzz_^(2)^, χ_zxx_^(2)^, and χ_xxz_^(2)^. χ_zxx_^(2)^ = χ_zyy_^(2)^ = χ_xzx_^(2)^ = χ_yzy_^(2)^, and χ_xxz_^(2)^ = χ_yyz_^(2)^ due to the *xy* plane being isotropic and the symmetry of the polarizability tensor. The rest of the elements are exactly zero except for large chiral environments.^26,27^ In the case of the water/air interface, the nonzero Imχ^(2)^ tensor components differ essentially by the total intensity, the relative intensity of the free O–H and hydrogen-bonded O–H regions, and the presence or absence of a shoulder on the lower-frequency side of the free O–H peak.^28^ Furthermore, the significantly small Im χ_yzy_^(2)^ spectrum compared with other polarization combinations is consistent with the experimental data.^29^

We proceed now with a systematic evaluation of the ML predictions of the VSFG spectra. First, we consider a PES given by the POLY2VS force field and focus on the impact of the different models of α and *P*. Panels a–c in Figure 1 show χ_xxz_^(2)^, χ_zzz_^(2)^, and χ_zxx_^(2)^, respectively. The ML-POLY-A and ML-POLY-B models are trained on POLY2VS reference data computed on bulk water and water cluster configurations, respectively (Table 1). As shown in Figure 1, both models have similar accuracy in their predictions of χ_xxz_^(2)^ and χ_zzz_^(2)^ and provide a semiquantitative agreement with the reference spectra. This is a rather surprising result since the ML-POLY-A model lacks information of interfacial water structures, whereas the ML-POLY-B model had only limited information on fully solvated water molecules. The prediction of χ_zxx_^(2)^ is considerably worse than those of the other two tensor components, with ML-POLY-A performing only slightly better than ML-POLY-B for the free O–H peak. By performing cross-predictions using *P*/α from the ML model and α/*P* from the reference, we identified the α predictions as the source of the observed inaccuracy (see Figure S15). This result highlights the limitation of using RMSE values and correlation plots as error estimators, as all components of α show a comparable accuracy.

**Figure 1:**
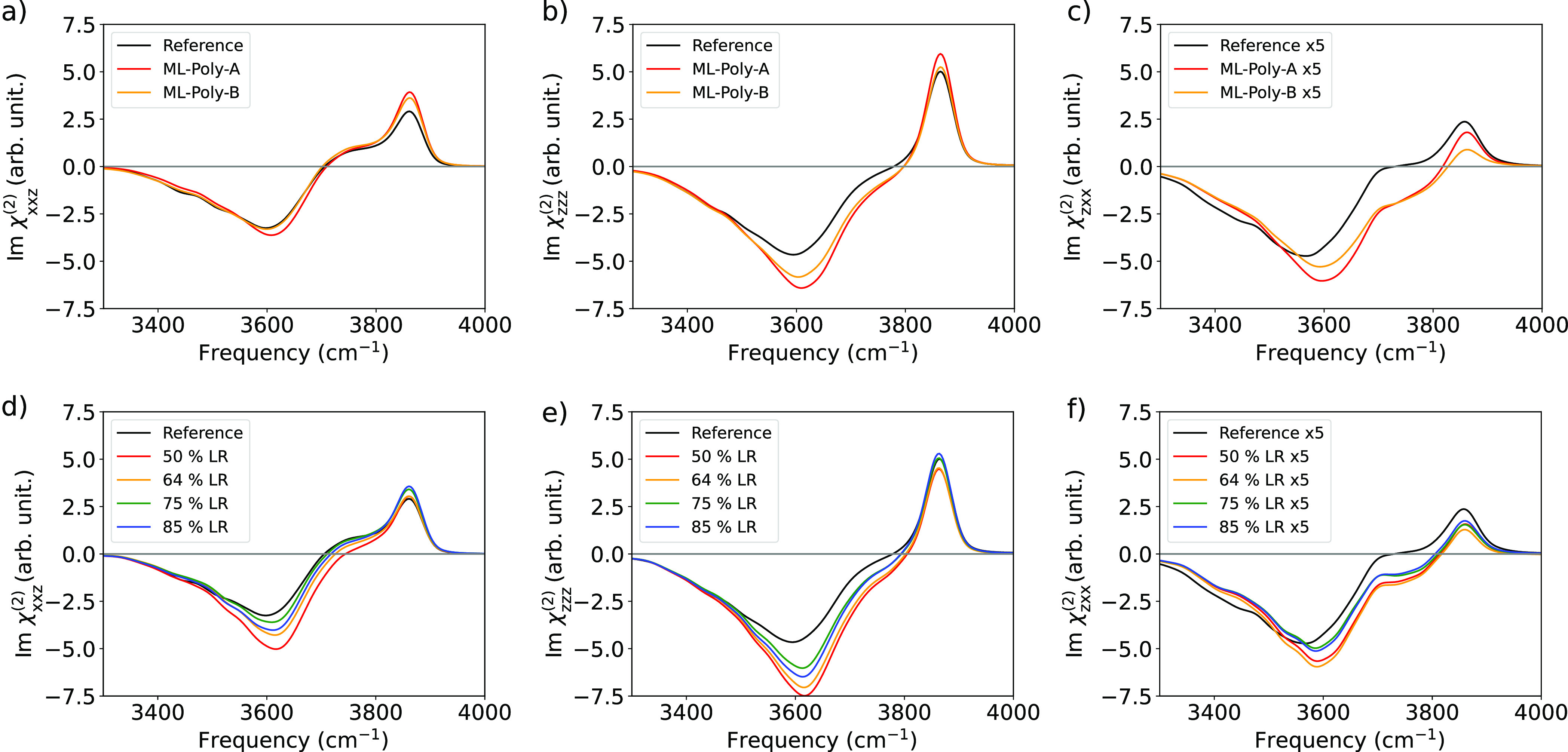
(a–c) Imaginary part of nonzero and independent χ^(2)^ components of the water/air interface using the POLY2VS model (reference) and ML-POLY-A and ML-POLY-B SA-GPR models. (d–f) Same as panels a–c but using ML-POLY-A augmented by different amounts of long-range (LR) contributions. χ_zxx_^(2)^ spectra are multiplied by a factor of 5 to ease visualization.

Electrostatic LR effects are known to play an important role at interfaces due to unbalanced interactions that can build up in the presence of a broken translation symmetry.^30^ The implications of using short-range models on structural properties of the water/air interface, such as orientations of water molecules or average density are well documented.^31,32^ ML models that include LR effects are also known to be more accurate.^30,33^ However, their possible impact on nonlinear spectroscopic responses, such as χ^(2)^, is less clear. To explore the magnitude of this effect, we trained a long-distance equivariant (LODE) model based on the local value of a atom-density potentials.^33^ In Figure 1d–f, we show the predictions using a combination of short-range (SR) and LR contributions, since the combined model normally delivers a superior accuracy compared to the individual ones. In particular, we considered different amounts of LR contributions in the range of 50%–85%. The best combination across all the tensor components is obtained for a 25/75 SR/LR model, but the performance is very similar to that of SR models. These results unequivocally demonstrate that LR effects on the descriptions of *P* and α have a marginal impact on the χ^(2)^ spectra.

Having established the suitability of the training set and the methodology to described *P* and α surfaces, we now consider the spectral changes induced by different *ab initio* PES. In Figure 2, we present the computed VSFG spectra using SA-GPR response surfaces trained on PBE (ML-PBE-A) and POLY2VS (ML-POLY-A) data and six different *ab initio* potential energy surfaces, namely, PBE-D3, BLYP-D3, revPBE-D3, HSE06, B3LYP, and revPBE0-D3. The D_2_O trajectories used for this analysis were available from a previous work.^34^ Due to the limited length of the simulations, it was necessary to neglect intermolecular terms in eq 1 to obtain a reasonable converged spectrum (see more details in the Supporting Information). Thus, the width of the negative band is poorly described due to the absence of intermolecular couplings.^14^ In all cases except for PBE-D3, the spectra do not show a positive signal below 2200 cm^–1^, in agreement with the latest measurements and simulations.^35,36^ For all the considered PESs, the ML-PBE-A model consistently overestimates (underestimates) the intensity of the free O–D peak (the hydrogen bonded (HB) O–D band), while the ML-POLY-A model predicts relative intensities in better agreement with the experiments. The better performance of ML-POLY-A is a direct consequence of the fact that the reference POLY2VS dipole and polarizability surfaces were fitted to reproduce CCSD/aug-cc-pVQZ, rather than DFT, reference values. The calculations with GGA exchange correlation (XC) functionals (PBE-D3, BLYP, and revPBE-D3) are artificially red-shifted in comparison to the results obtained with hybrid ones (HSE06, B3LYP, and revPBE0-D3) in agreement with previous approximations based on the ssVVCF methodology^34^ and results on bulk water.^37,38^ Inclusion of nuclear quantum effects (NQEs) is known to induce a frequency red-shift when compared to the corresponding classical nuclei counterpart spectra.^39,40^ Thus, the frequency agreement between the revPBE-D3 PES and the experimental data is a fortuitous error compensation.^38^

**Figure 2:**
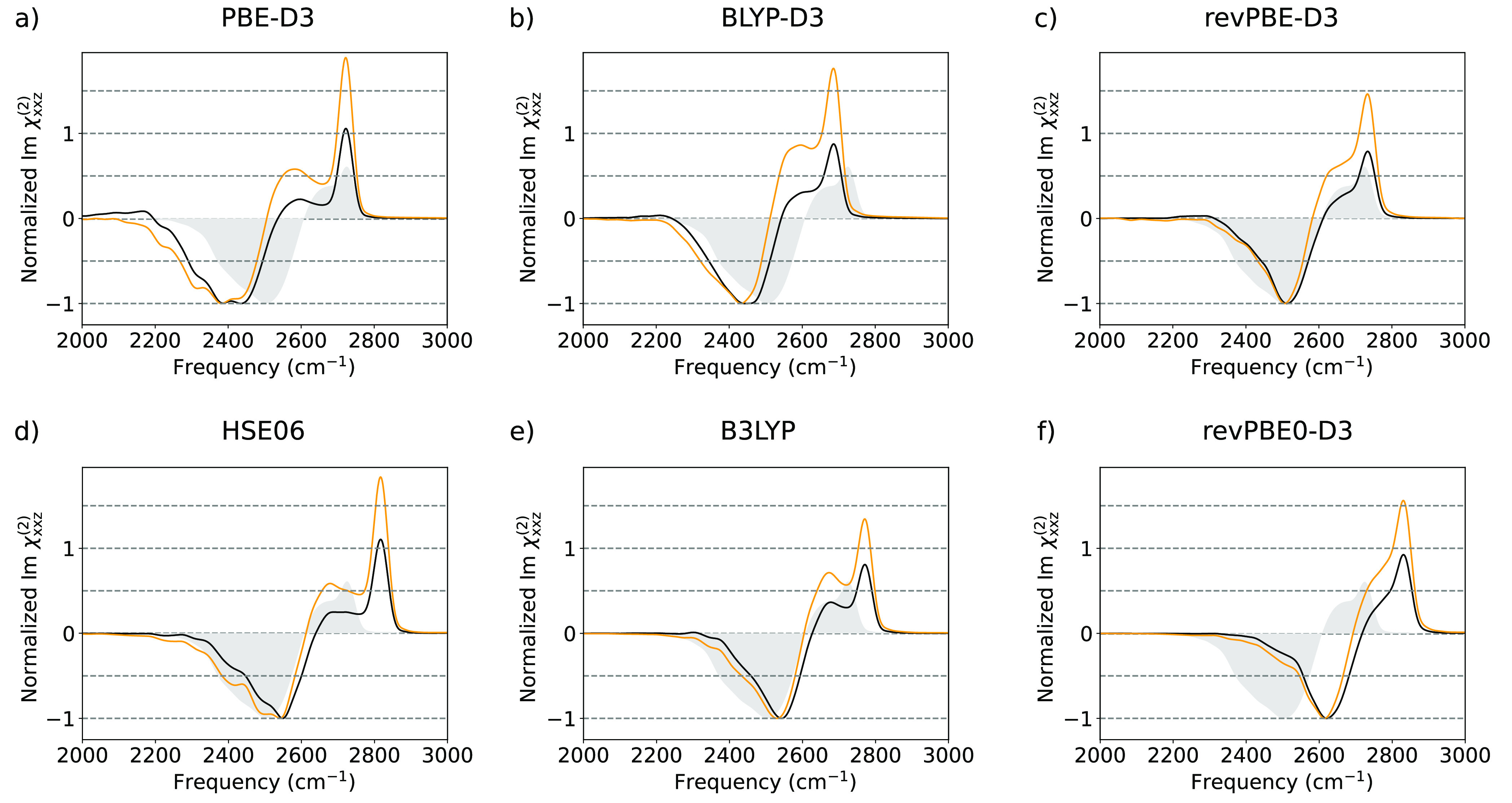
Simulated SFG spectra (χ_*xxz*_^(2)^) of the water/air interface using trajectories obtained with *ab initio* PES and ML-PBE-A and ML-POLY-A are depicted by orange and black lines, respectively. Experimental spectra, corrected with Fresnel factors, assuming the Lorentz model for the interfacial dielectric constant, are depicted as gray shaded area.^44^

The intramolecular vibrational coupling of water molecules in which one O–D is a hydrogen-bond donor and the other one is free has a distinctive spectral feature associated with a shoulder on the free O–H peak. This shoulder has been assigned to the asymmetric stretching mode of interfacial water molecules,^41,42^ and contributions to this shoulder from water molecules forming two hydrogen bonds have been shown to be minor due to cancellation of inter- and intramolecular contributions.^12^ PBE-D3, BLYP-D3, HSE06, and B3LYP spectra present a shoulder so separated from the free O–D peak that it can be regarded as a separate peak. In contrast, this spectral feature is correctly displayed by the revPBE0-D3 and revPBE-D3 spectra. Moreover, the differences in performance for revPBE0-D3 and revPBE-D3 are relatively small and mainly impact the intensity of the free O–D peak and an overall frequency shift. These results show that even hybrid functionals, such as HSE06 and B3LYP, considerably overestimate the H-bond strength, unlike in bulk water.^43^

Finally, we show fully-ML predictions for the Im χ_xxz_^(2)^ spectra for D_2_O/air at 300 K in Figure 3 (Re χ_xxz_^(2)^ is reported in the Supporting Information). We focus on HDNNP trained on revPBE0-D3^45^ and revPBE-D3^46^ XC functionals since this level of theory is known to describe the structure and dynamics of liquid water accurately.^38^ The overall agreement between the spectra for each XC functional is remarkable when considering the computational costs associated with each type of calculation. The revPBE0-D3 HDNNP result shows a free O–D peak with a more pronounced shoulder and a blue-shift of 33 cm^–1^ with respect to the DFT prediction presented previously in Figure 2. Conversely, the revPBE-D3 HDNNP spectra show a less pronounced free O–D peak shoulder and a red-shift of −14 cm^–1^. Both HDNNPs were trained without an explicit treatment of LR interactions, which are known, as mentioned previously, to be responsible for the net orientation of the water molecules in the bulk region. We believe that these artifacts are responsible for the (small) discrepancies with the fully *ab initio* spectra. In particular, the spurious additional orientation of water molecules, which happens in opposite directions for revPBE-D3 and revPBE0-D3 HDNNPs, as shown in Figure S17, might be responsible for increasing and decreasing the splitting between free and HB O–D vibrations of water molecules at the topmost layer. However, for all tested cases, irrespective of the PES, the theoretical predictions overestimate the intensity ratio between the free O–D and the hydrogen-bonded band. This underestimation has been reported by Paesani and co-workers when induction effects arising from interactions between individual molecules are neglected.^47^ Since SA-GPR can capture local induction effects, the large discrepancy is attributed to the quality of the underlying reference data. Once more, the ML-POLY-A model outperforms ML-PBE-A. For the revPBE0-D3 case, we also tested the ML-PBE0-A model, in which the polarization was fitted to PBE0 reference data but the polarizability was kept at the PBE level. While it represents an improvement with respect to the ML-PBE-A model, particularly in the description of free O–D shoulder, it still underperforms compared to the ML-POLY-A results.

**Figure 3:**
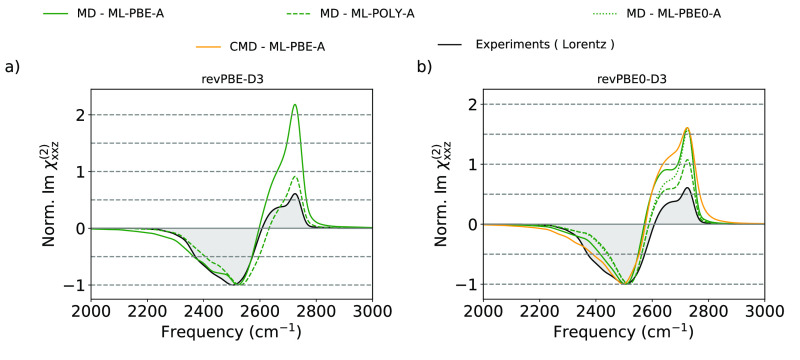
Normalized χ_*xxz*_^(2)^ spectra of the D_2_O/air interface at 300 K. Simulated spectra using revPBE-D3 (left) and revPBE0-D3 (right) XC functionals. Classical MD simulations using HDNNPs are presented by solid (ML-PBE-A), dashed (ML-POLY-A), and dotted (ML-PBE0-A) green lines. CMD simulations are depicted with solid orange lines. Experimental spectra are depicted as black solid lines with gray shading areas. Spectra were rigidly shifted to match experimental spectra. The values of the frequency shifts for revPBE0-D3 (revPBE-D3) are MD-HDNNP −138 cm^–1^ (+4 cm^–1^) and CMD-HDNNP −57 cm^–1^. Experimental spectra are corrected by the appropriate Fresnel factors, assuming the Lorentz model (black dotted lines) for the interfacial dielectric constant.^44^ To allow a visual comparative analysis, the spectra are normalized such that the HB O–D band has an intensity of unity.

So far, we have analyzed simulations where the nuclei were assumed to behave classically, and the spectra have been rigidly red-shifted to account for the ignored NQEs. In the right panel of Figure 3, we show the results obtained with centroid molecular dynamics (CMD), which is a well-established method to simulate the vibrational spectra of condensed phase systems at room temperature including an approximate description of NQEs.^48,49^ CMD is based on the path integral formulation of quantum mechanics,^50^ and in this method, the nuclei are evolved according to classical equations of motion on the so-called centroid potential of mean force. NQEs induce a broadening of the free O–D peak and HB-band and a red-shift of 81 cm^–1^ with respect to the corresponding classical nuclei simulations. However, the CMD spectrum is still blue-shifted by 57 cm^–1^ with respect to the experimental result. We also obtained the VSFG spectra using the thermostated ring polymer molecular dynamics (TRPMD)^51^ method which is known to deliver more accurate frequencies than CMD at 300 K since it does not suffer from the curvature problem^49,52−54^ (see Figure S19). By comparing the CMD and TRPMD results, we deduce that the curvature problem is responsible for an additional 10 cm^–1^ red-shift. We also compared with the latest refinement of the TRPMD method that employs thermostats based on the generalized Langevin equation that has been specifically designed to mitigate some of the artifacts associated with the original TRPMD formulation,^55^ arriving to the same conclusion (see Figure S19). Thus, by accounting for the 33 cm^–1^ blue-shift induced by the lack of LR effects in the HDNNP discussed previously, we conclude that the error introduced by DFT in the revPBE0-D3 XC functional approximation is within the theoretical limit of quantum-statistical classical-dynamics methods which overestimate high-frequency modes by about 50 cm^–1^.^53,54^

The combination of two different types of ML algorithms, namely, HDNNPs and SA-GPR, has allowed us to describe the water/air interface and all the components (polarizations) of second-order response from first principles at an affordable computational cost. The ML models were trained in a general fashion, without employing specific information on the nature of the system, such as physical constraints designed for water, mapping models for water,^56^ or the use of a Δ-ML procedure based on an available surrogate model.^57^ While at the moment force fields tailored to describe water outperform the presented results,^12,28,44^ we stress that the procedure and strategy presented are directly applicable to any reference data set, to larger systems, and most importantly, to more complex and reactive interfaces.

Several other ML approaches with varying architectures have been reported in the last five years to predict molecular dipole moments^58−61^ and linear vibrational spectroscopy.^62−64^ However, attempts to simulate VSFG spectroscopy have been lagging behind, most likely due to the destructive interference of the signal, which made those approaches inappropriate for this target. While the strategy presented here could be easily applied to other kernel regression models that use local representations, it remains to be seen if similar ideas could be implemented on neural network architectures.

We evaluated the impact of the different components that are involved in the calculation of the VSFG spectra. By comparing the performance of different XC functionals, we found that the PES is best described by the revPBE-D3 and revPBE0-D3 XC functionals. These results add to the existing evidence confirming that XC functionals constrained by exact functional conditions with suitable dispersion corrections, such as revPBE-D3 and revPBE0-D3, deliver excellent performance in the description of water.^38^ Since the discrepancies between the theoretical and the experimental spectra are larger than the training error of the SA-GPR models, we attribute the largest source of error to the reference data used to describe the *P* and α surfaces. A further study using electronic structure methods beyond DFT to obtain more accurate *P* and α surfaces is urgently needed. We note that the modeling of the VSFG spectra described by eq 1 is derived in the electric dipole approximation.^5^ We believe that applying the current methodology to a more accurate reference data set would finally resolve existing controversies related to, for example, the relevance of quadrupole contributions in the water bending mode.^65,66^

We showed that the off-diagonal components of the α tensor for water are more difficult to learn due to their smaller magnitude when compared to the diagonal ones, which leads to a poorer description of certain χ^(2)^ matrix elements. Since spherical components mix together diagonal and off-diagonal elements, it is not possible to train the off-diagonal elements exclusively with current SA-GPR implementations. Future efforts will be directed in this direction, in the development of machine learning models to predict Wannier centers,^67^ atomic polar tensors,^68^ and in the inclusion of explicit LR in the description of the PES.

Two different methods were considered to describe the time evolution of the nuclei: MD and CMD. CMD is appropriate to describe the D_2_O/air interface and predicts a peak position that is in good agreement with experiments. However, the curvilinear motion of dangling O–D in D_2_O shows an extremely broad feature,^47,69^ which calls for the use of other approaches which do not suffer from the curvature problem of CMD nor the broadening problem of TRPMD. The recent method proposed by Musil et al. seems a promising approach to tackle this issue.^70^ We note, however, that none of these can describe accurately the Fermi resonance contributions.^44,53,71,72^

In summary, the work presented here sets a new standard for atomistic simulation of nonlinear spectroscopies of condensed phases. While we have focused on the second-order response which is related to VSFG spectroscopy, the approach presented here paves the way to *ab initio* simulations of 2D-VSFG, 2D-IR, 2D THz-Raman, and 2D ThZ-IR-Vis spectroscopies.^73−75^ We expect that applications of the presented strategy would bring important atomistic insights into the properties of aqueous interfaces at metallic and biological surfaces^76^ in solution and under confinement.^77^

## Supporting Information Available

Detailed description of the VSFG calculations, learning curves, error analysis, and further validation tests (PDF)
Transparent Peer Review report available (PDF)
