# Supplementary material for: Fully First-Principles Surface Spectroscopy with Machine Learning
Source: J Phys Chem Lett. 2023 Sep 6;14(36):8175–82. doi: 10.1021/acs.jpclett.3c01989 (PMC10510433; doi:10.1021/acs.jpclett.3c01989)
Supplement: Supplementary file 1 — jz3c01989_si_001.pdf [file jz3c01989_si_001.pdf]

Fully First-Principles Surface Spectroscopy with Machine Learning

Yair Litman,[1,2,a)] Jinggang Lan,[3,4] Yuki Nagata,[2] and David M. Wilkins[5,b)]

[1)] *Yusuf Hamied Department of Chemistry, University of Cambridge, Lensfield Road, Cambridge, CB2 1EW, United Kingdom*

[2)] *Max Planck Institute for Polymer Research, Ackermannweg 10, 55128 Mainz, Germany*

[3)] *Department of Chemistry, New York University, New York, NY, 10003, USA*

[4)] *Simons Center for Computational Physical Chemistry at New York University, New York, NY, 10003, United States*

[5)] *Centre for Quantum Materials and Technology, School of Mathematics and Physics, Queen's University Belfast, Belfast BT7 1NN, Northern Ireland, United Kingdom*

[a)] Electronic mail: yl899@cam.ac.uk

[b)] Electronic mail: d.wilkins@qub.ac.uk

METHODS

Molecular Dynamics Simulations and Vibrational sum frequency generation (VSFG) calculations

The water/air interface was modelled using a water slab made of 160 water molecules contained in a 16.63×16.63×44.14 Å^3 simulation box. The simulations were carried out using the i-PI code[1] connected to the LAMMPS package[2,3]. using the neural network potential reported in Ref.[4] and[5]. Unless specified otherwise, the results presented with classical nuclei molecular dynamics (MD) were obtained as an average of 20 independent 100 ps trajectories in which the initial configurations were obtained by 100 ps thermalization runs at 300K using the stochastic velocity rescaling thermostat[6] with a time constant of 200 ps. The partially adiabatic CMD simulations were carried out using the same setup as reported elsewhere[7]. In all cases, the configurations were saved every 4 fs in the production runs for a posterior calculation of P and α through the symmetry-adapted Gaussian process regression (SA-GPR) or POLY2VS[8] models. The *ab initio* trajectories were obtained from a previous work[9].

By combining Eq. 3 into Eq. 1 of the main text, we obtain

$$\chi^{(2),R}_{pqr}(\omega_{\mathrm{IR}}) = i\int_0^{\infty} dt e^{-i\omega_{\mathrm{IR}}t} \sum_{\gamma_1,\gamma_2} \langle g(z_{\gamma_2}, z_1, z_2)\alpha_{pq}(\mathcal{X}_{\gamma_1}, t)P_r(\mathcal{X}_{\gamma_2}, 0)\rangle, \tag{1}$$

where $\alpha_{pq}(\mathcal{X}_{\gamma_1}, t)$ is the polarizability of the molecule γ_1 at time t and $P_r(\mathcal{X}_{\gamma_2}, 0)$ is the molecular dipole of the molecule γ_2 at time 0. The surface normal is assumed to be parallel to the z-axis and z_{γ_2} corresponds to z coordinate of the γ_2 water molecule. The results obtained from the *ab initio* trajectories (Fig. 2 of the main text) were calculated by considering only the terms $\gamma_1 = \gamma_2$ in the sum of Eq. 1. This approximation was necessary due to the limited length of the trajectories. For all the other results, the sum was evaluated for all pairs of water molecules separated by less than 4 Å. This cutoff value provides a converged spectra, see for example[10]. Unless specified, we use $z_1 = 4$ Å and $z_2 = 2$ Å as reported in Ref.[11], and before the calculation of the Fourier transform the correlation function was convoluted with a Hahn window such that it decays to zero after 1.0 ps. Experimental data were processed consistently.

Hybrid DFT Calculations

The hybrid PBE0 calculations were performed using the CP2K program[12]. Molecular orbitals of the valence electrons were expanded in the TZV2P basis sets[13], while atomic core electrons were described through Goedecker-Teter-Hutter (GTH) pseudopotentials corresponding to the PBE functional[14,15]. Exact exchange integrals were calculated within the auxiliary density matrix method (ADMM) approximation[16]. In addition, the truncated Coulomb operator[17] has been applied for the exchange calculations with the cutoff radius approximately equal to half the length of the smallest edge of the simulation cell, together with the Schwarz integral screening with the threshold of 10^{-10} a. u. The cutoff for the auxiliary plane waves was 800 Ry.

The total electric dipole moment μ is evaluated using the Berry phase scheme[18,19]. With a side length L and considering only the Γ point in the Brillouin zone, each component of the electric-dipole moment is then

$$\mu = \frac{e}{2\pi} \mathrm{Im}\ \ln\ \det \mathbf{S}, \tag{2}$$

where the matrix $\mathbf{S}$ is defined using the Kohn-Sam orbitals ϕ_n, and

$$S_{n,m}(x) = \int_L \phi_n^*(x) \exp\left[-i\frac{2\pi}{L}x\right] \phi_m(x) dx. \tag{3}$$

The polarizability α is calculated using the finite difference method, where a periodic electric field is applied in a given direction.

$$\alpha = \frac{d\mu}{dE}. \tag{4}$$

SYMMETRY-ADAPTED GPR MODELS

For each dataset, SA-GPR models were built for the polarization $\boldsymbol{P}$ and the $\lambda = 0$ and $\lambda = 2$ spherical tensor components of the polarizability[20,21]. We used the hyperparameters of Ref.[22], which were found to perform well for bulk water. The supplementary information includes scripts for the training procedure, in which the Cartesian tensor properties of the training set frames are converted to spherical tensors, the λ-SOAP descriptors of Ref.[20] are calculated for each local environment in every training configuration and these are combined to give λ-SOAP kernels, which are used to train separate models for each spherical tensor component. The supplementary information includes a script that shows in detail how this

process works. In each case, the full data set was split 80%:20% with the smaller set used to test the hyperparameters and ensure that the model was not overfitting. The final model for each set was trained on the entire set. The SI also contains each of the models developed and a script that can be used to apply them. The mixed short-range/long-range models are built using kernels of the form,

$$\boldsymbol{K}^{\lambda}(\mathcal{X},\mathcal{X}') = w_{\mathrm{S}}\boldsymbol{K}^{\lambda}_{\mathrm{SR}}(\mathcal{X},\mathcal{X}') + w_{\mathrm{L}}\boldsymbol{K}^{\lambda}_{\mathrm{LR}}(\mathcal{X},\mathcal{X}'), \tag{5}$$

where $\boldsymbol{K}^{\lambda}(\mathcal{X},\mathcal{X}')$ is the total spherical tensor kernel of order λ between two local environments $\mathcal{X}$ and $\mathcal{X}'$, $\boldsymbol{K}^{\lambda}_{\mathrm{SR}}$ is the (short-ranged) λ-SOAP kernel[20], $\boldsymbol{K}^{\lambda}_{\mathrm{LR}}$ the (long-ranged) λ-LODE kernel[23] and $w_{\mathrm{S}} + w_{\mathrm{L}} = 1$

For the ML-PBE-A model, we used the bulk water data of Ref.[20], comprising 1000 frames of 32 molecules, with polarizations and polarizabilities computed using the PBE functional and ultrasoft pseudopotentials. For these 1000 frames, the polarizations were recomputed at the PBE0 level to produce the ML-PBE0-A set. These configurations were also used for the ML-POLY-A set, with both the polarization and the polarizability recomputed with the POLY2VS model. The ML-POLY-B models were produced using a set of water clusters: water n-mer configurations (with $n = 1, \ldots, 6$) were taken from the training set used in Ref.[24]: 10,000 clusters were used for each value of n, with the POLY2VS model used to calculate the dipole moment $\boldsymbol{\mu}$ and $\boldsymbol{\alpha}$. The predictions of ML-POLY-B give atom-centred dipole moments, which can be summed up to give a prediction of the total polarization of a frame.

The accuracy of SA-GPR models for the polarizability is given by the root mean squared error (RMSE),

$$\mathrm{RMSE}_{\alpha} = \sqrt{\frac{1}{N}\sum_{i=1}^{N} \|\boldsymbol{\alpha}_{\mathrm{calc},i} - \boldsymbol{\alpha}_{\mathrm{pred},i}\|_2^2}, \tag{6}$$

where $\| \cdots \|_2$ is the Frobenius norm, N the number of members of the training set, $\boldsymbol{\alpha}_{\mathrm{calc},i}$ the calculated polarizability for the ith testing point and $\boldsymbol{\alpha}_{\mathrm{pred},i}$ its predicted polarizability. For the polarization we use,

$$\mathrm{RMSE}_{P} = \sqrt{\frac{1}{N}\sum_{i=1}^{N} \||\boldsymbol{P}|_{\mathrm{calc},i} - |\boldsymbol{P}|_{\mathrm{pred},i}\|_2^2}, \tag{7}$$

with $\|\boldsymbol{P}\|_{\mathrm{calc},i}$ the magnitude of the calculated polarization of testing point i and $\|\boldsymbol{P}\|_{\mathrm{pred},i}$ its predicted value.

Training of SA-GPR models

The learning curves for polarization (P) and polarizability (α) for ML-POLY-A and ML-POLY-B models are presented in Fig. S1 and S2, respectively. The learning curves for ML-PBE-A can be found elsewhere[22] and P learning curve for ML-PBE0-A is presented in Fig. S3. In Fig. S4 (S6) and S5 (S7), we present the per-component correlation plots for total α and P predictions for the ML-POLY-A (ML-POLY-B) model. All these figures demonstrate that SA-GPR can handle cluster and bulk structures equally well. Analogous correlation plots for the ML-PBE-A and ML-PBE0-A models are shown in Fig. S8, S9, and S10, respectively.

Since the POLY2VS force-field uses a molecular decomposition of the P, and SA-GPR utilizes an atomic decomposition underneath, it is possible to analyze how well the molecular quantities are being represented. In Fig. S11 and S12, we present the corresponding plots for the molecular predictions. The errors obtained for molecular quantities are much larger than the ones obtained for the total quantities. This fact is not surprising since our models are trained on real observables (i.e. total P and total α) and we are not using any physical constraints on our models beyond the symmetry considerations.

In Fig. S13 and Fig. S14, we present the absolute percentage errors of the molecular predictions for ML-POLY-A and ML-POLY-B models, respectively, as a function of distance from the interface in the same setup used to compute the VSFG spectra, i.e. water slab structures. Note that these structures are not included in the training sets. Both models show similar performance, with ML-POLY-B performing slightly better, even though ML-POLY-A was trained on bulk structures exclusively, and surprisingly the predictions are more accurate in the vicinity of the interface. While the error distributions for the diagonal components of α are relatively narrow around the mean value, the off-diagonal elements show a more uniform distribution with errors up to 100%. We believe that the larger errors in the latter case are due to the smaller relative values of the off-diagonal elements with respect to the diagonal ones. The polarization components show a better performance than the off-diagonal elements but worse than the diagonal elements of α. Note that, as it is shown in the main text (and in Fig. S15 below), all the $\chi^{(2)}$ components are predicted satisfactorily except $\chi^{(2)}_{\mathrm{zxx}}$ which utilizes one of the off-diagonal components of α.

COMPUTATIONAL COST

The computational cost associated with the simulation of the VSFG spectra using the presented machine learning (ML) approach can be divided into four parts: i) Obtaining the reference data ($C_{\text{ref-data}}$), ii) Training the ML model (C_{train}), iii) Performing the molecular dynamics simulations (C_{MD}), and iv) Evaluation of the polarization and polarizability ($C_{P,\alpha}$). The cost of point ii) is negligible when compared to the other points so it is not considered further in this analysis. Furthermore, point i) can be obtained from a single 20 ps long molecular dynamics trajectory[25], which is 1000 times shorter than the trajectory length required to compute a VSFG spectrum. If one is interested in computing just a single VSFG spectra, $C_{\text{ref-data}}$ becomes the determining factor of the total cost. This represents a saving of three orders of magnitude when compared to direct ab initio simulations. On the contrary, if one is interested in obtaining spectra at different conditions, such as at slightly different temperatures, various isotopic mixtures, or using different methods to include nuclear quantum effects NQEs, then C_{MD} and $C_{P,\alpha}$ become the bottleneck. However, with the current implementations, the computational cost of the former contribution is 100-200 times smaller. Finally, the evaluation of the polarization and polarizability for a single configuration using the SA-GPR model and direct ab initio calculations have a computational cost of 0.01 CPU hours and 1000 CPU hours (Intel® Xeon® E5-2690 v3 @ 2.60GHz), respectively. In this scenario, the computational saving goes up to a factor of 10^5.

ADDITIONAL FIGURES

In Fig. S15, we present calculations for the imaginary part of $\chi^{(2)}$ using GPR and POLY2VS α-P surfaces using trajectories obtained with POLY2VS potential energy surface (PES). In Fig. S16, we compare the predicted spectra using different PES but the same POLY2VS α-P surfaces using different cutoff values (see Eq. 1). In Fig. S17, we compare the density and orientation profiles obtained with *ab initio* and neural-network PES. In Fig. S18 we present the real part of $\chi^{(2)}$ corresponding to the simulations shown in Fig. 3 in the main text. Finally, in Fig. S19 we present imaginary part of $\chi^{(2)}$ obtained using three different methods to propagate the nuclei: classical nuclei molecular dynamics (MD), thermostated ring polymer molecular dynamics (TRPMD)[26,27] and centroid molecular dynamics

(CMD)[28,29].

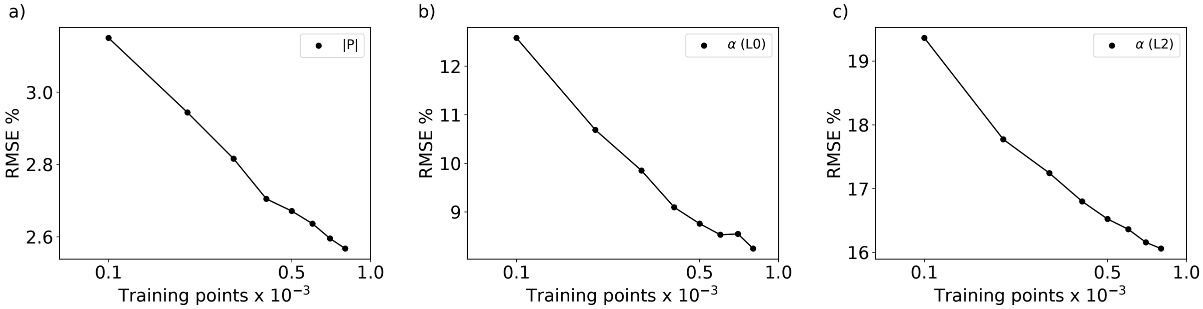

FIG. S1: Learning curve of total polarization (a), $L0$ component of α (b), and $L2$ component of α for ML-POLY-A model

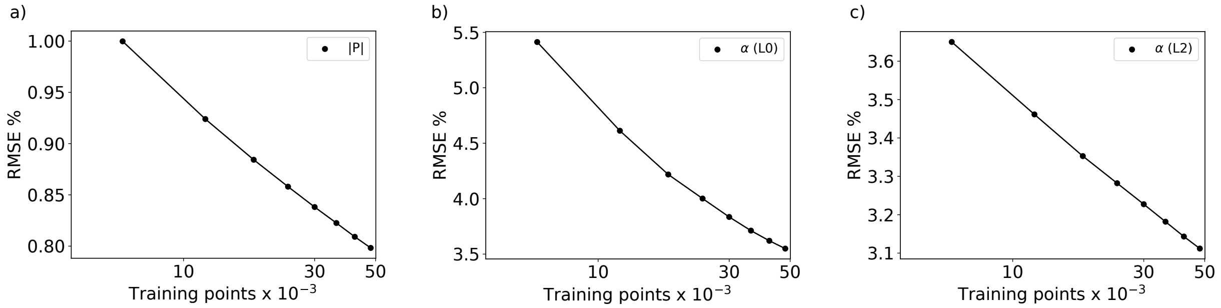

FIG. S2: Same as S1 for ML-POLY-B model

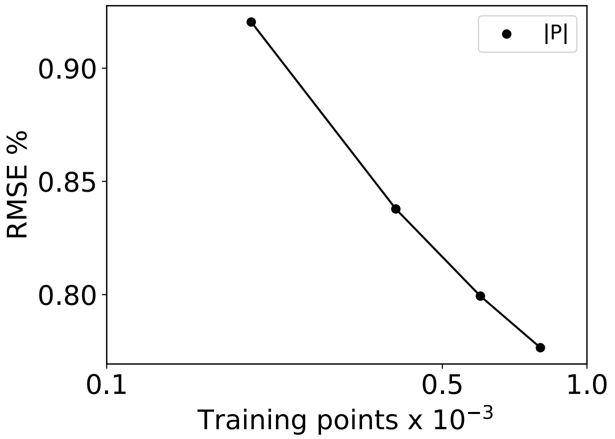

FIG. S3: Learning curve of total polarization fol ML-PBE0-A model.

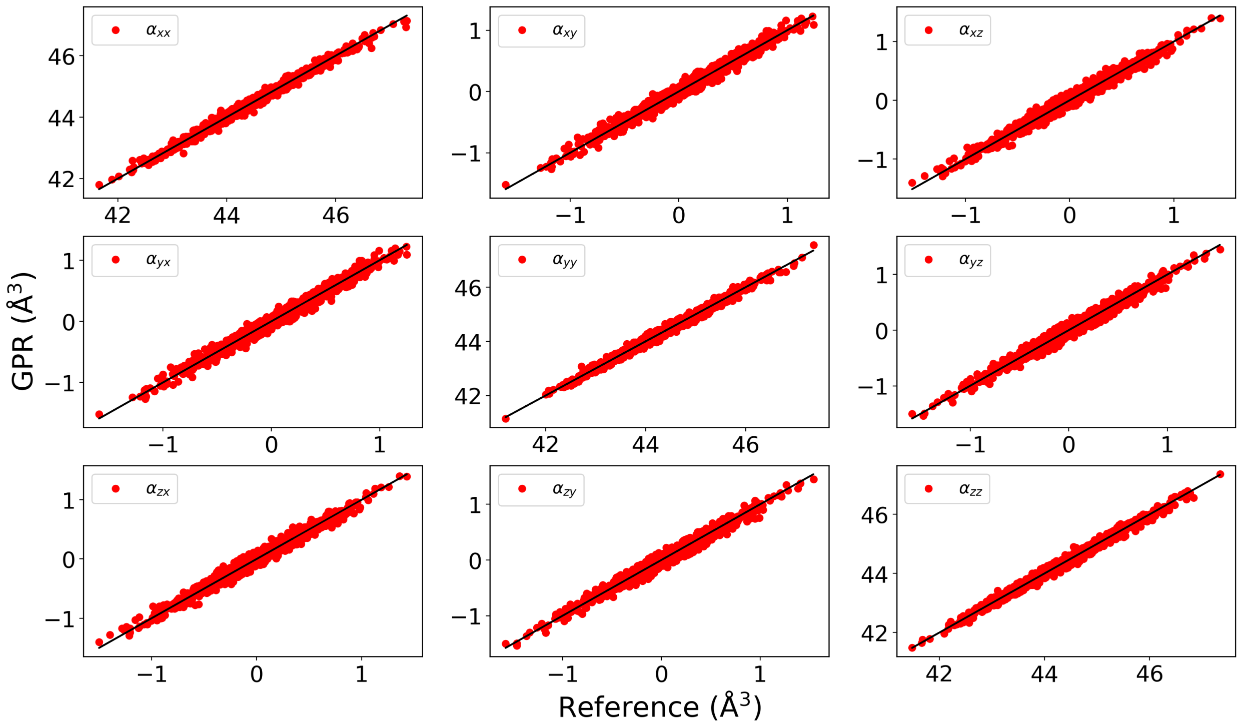

FIG. S4: Prediction of total α components for ML-POLY-A model.

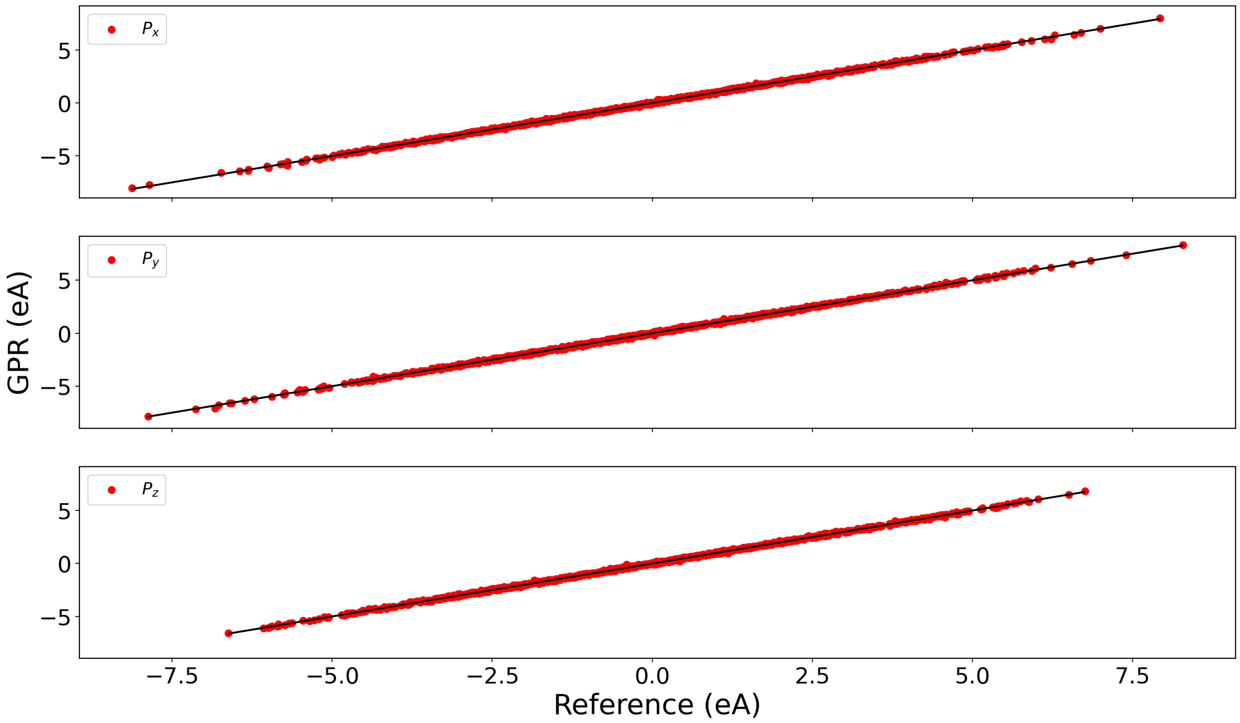

FIG. S5: Prediction of total P for ML-POLY-A model.

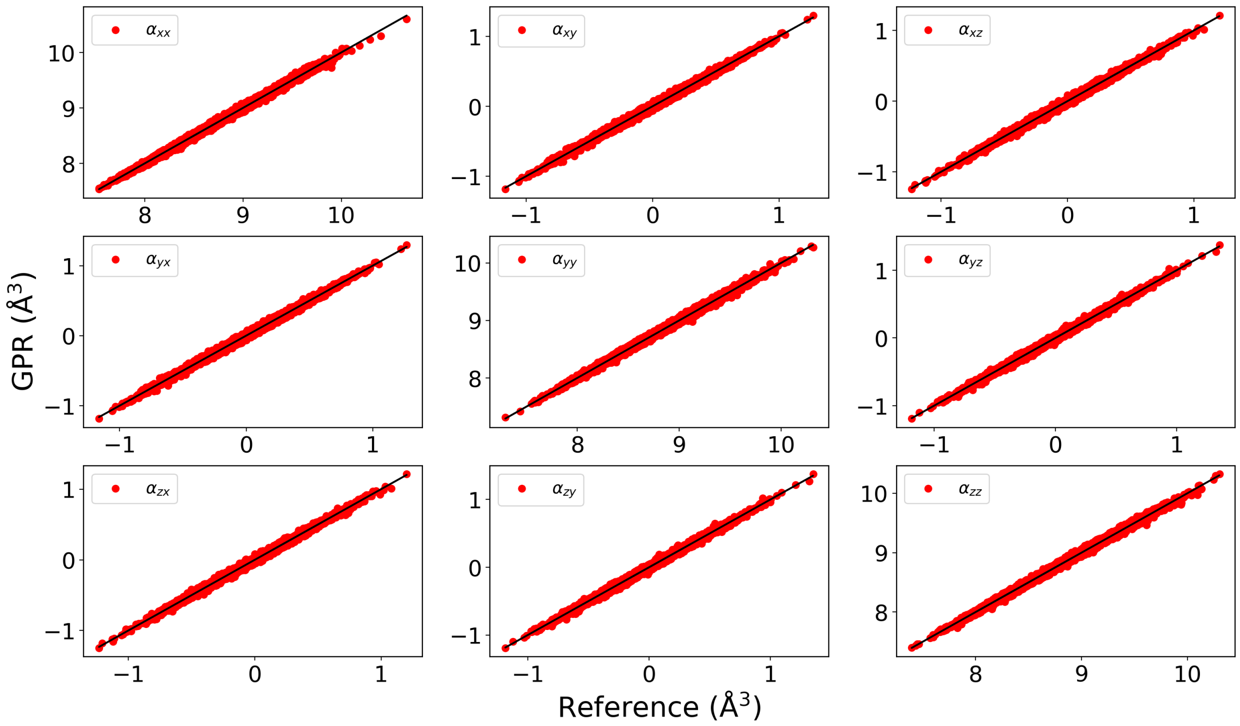

FIG. S6: Prediction of total α components for ML-POLY-B model.

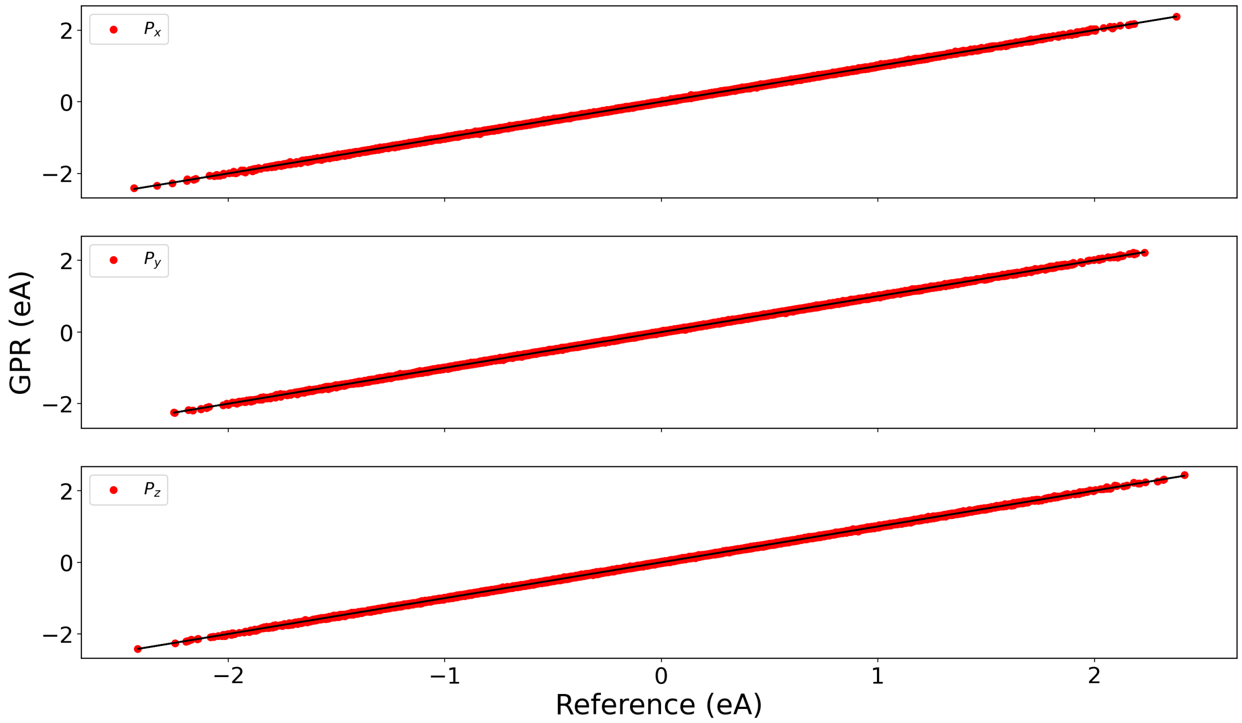

FIG. S7: Prediction of total P for ML-POLY-B model.

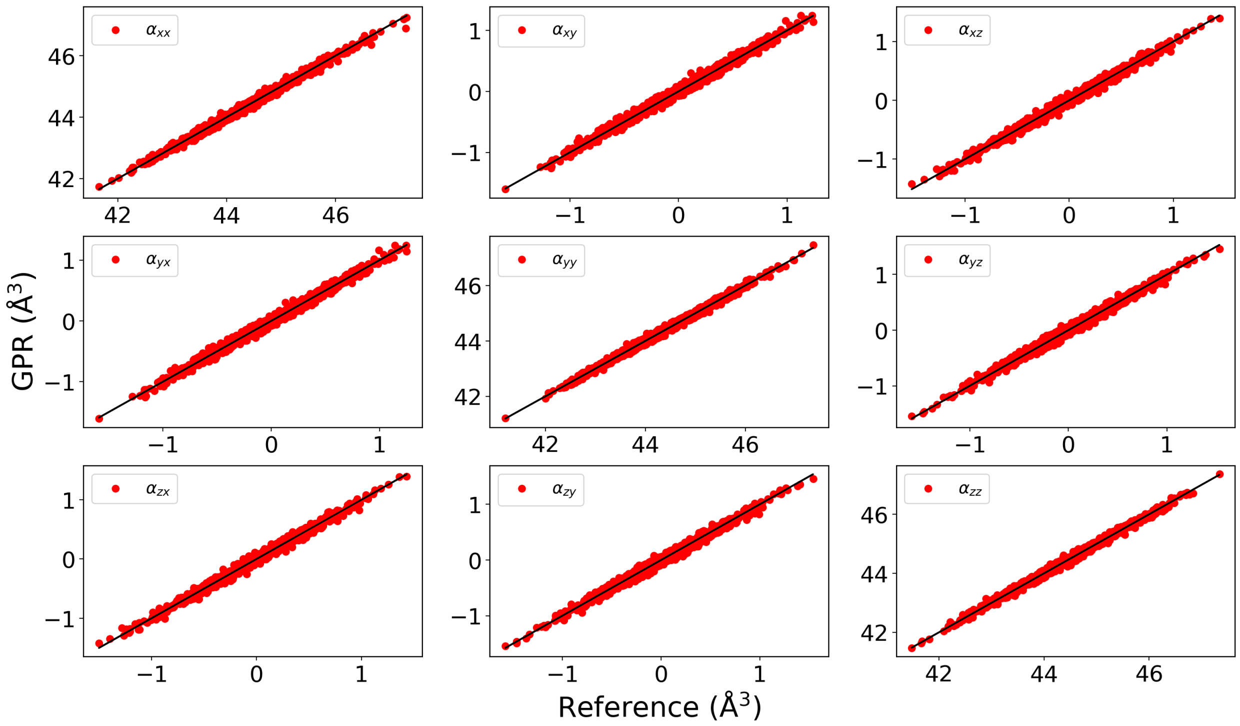

FIG. S8: Prediction of total α for ML-PBE-A model.

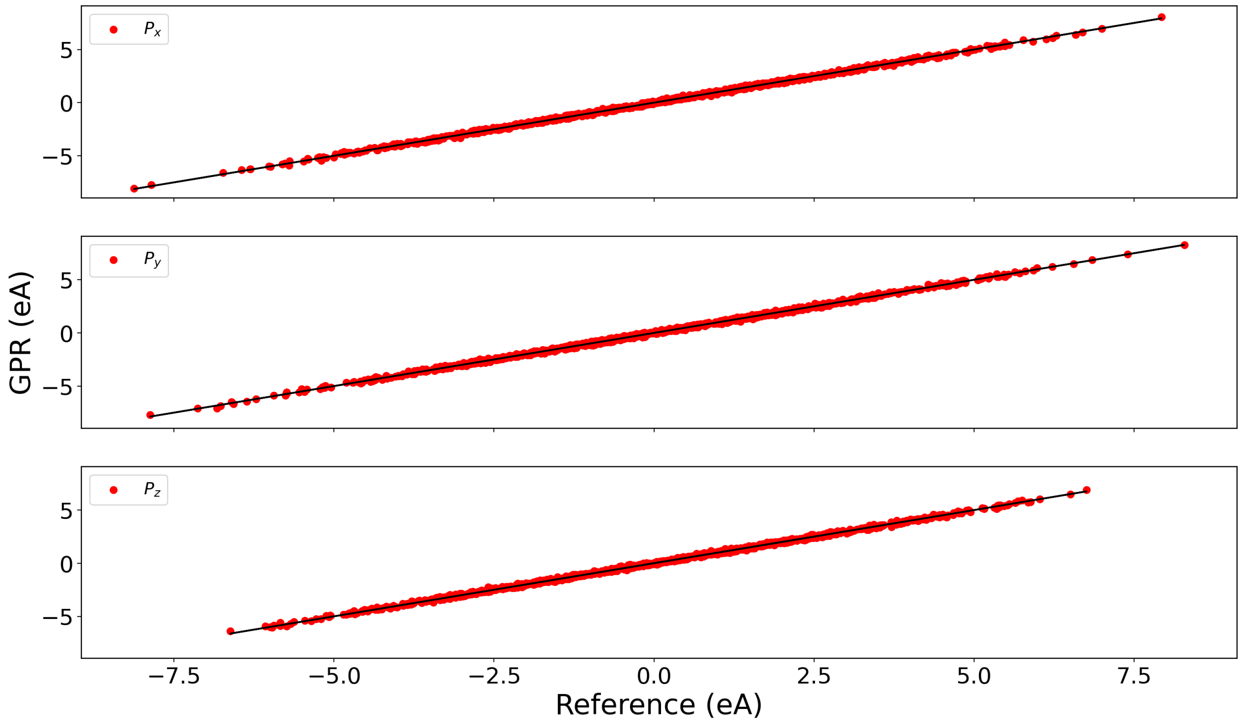

FIG. S9: Prediction of total P for ML-PBE-A model.

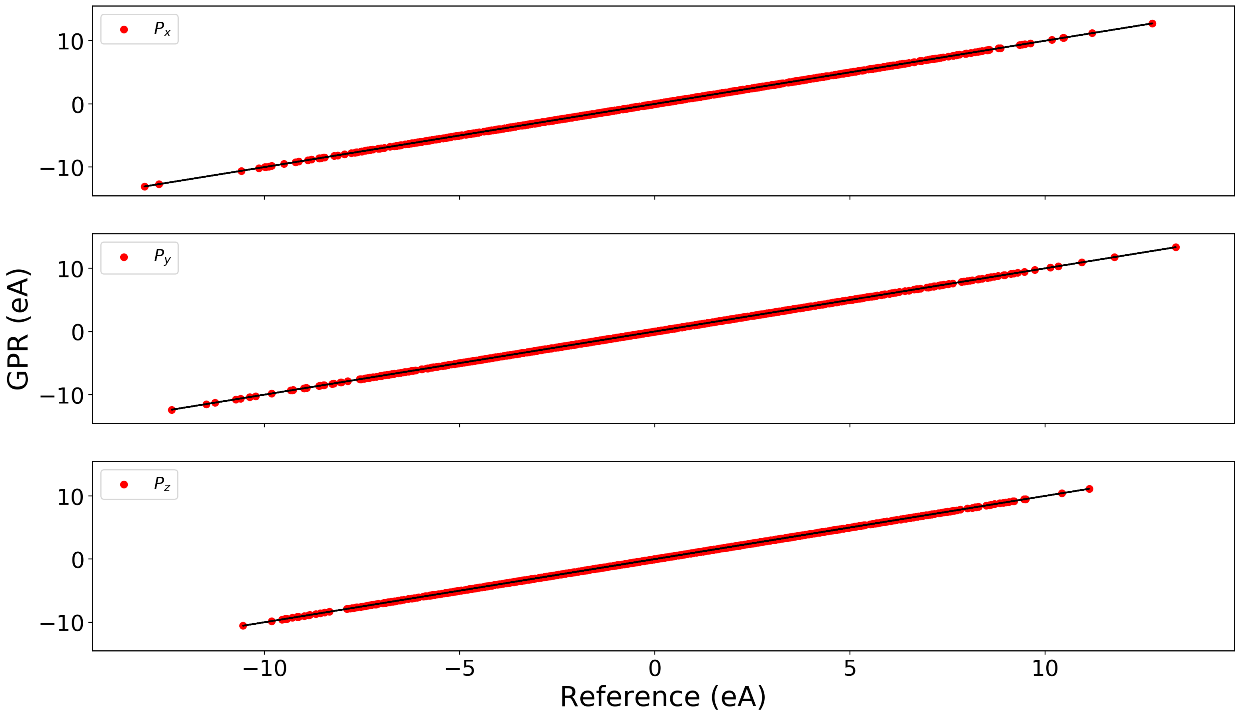

FIG. S10: Prediction of total P for ML-PBE0-A model.

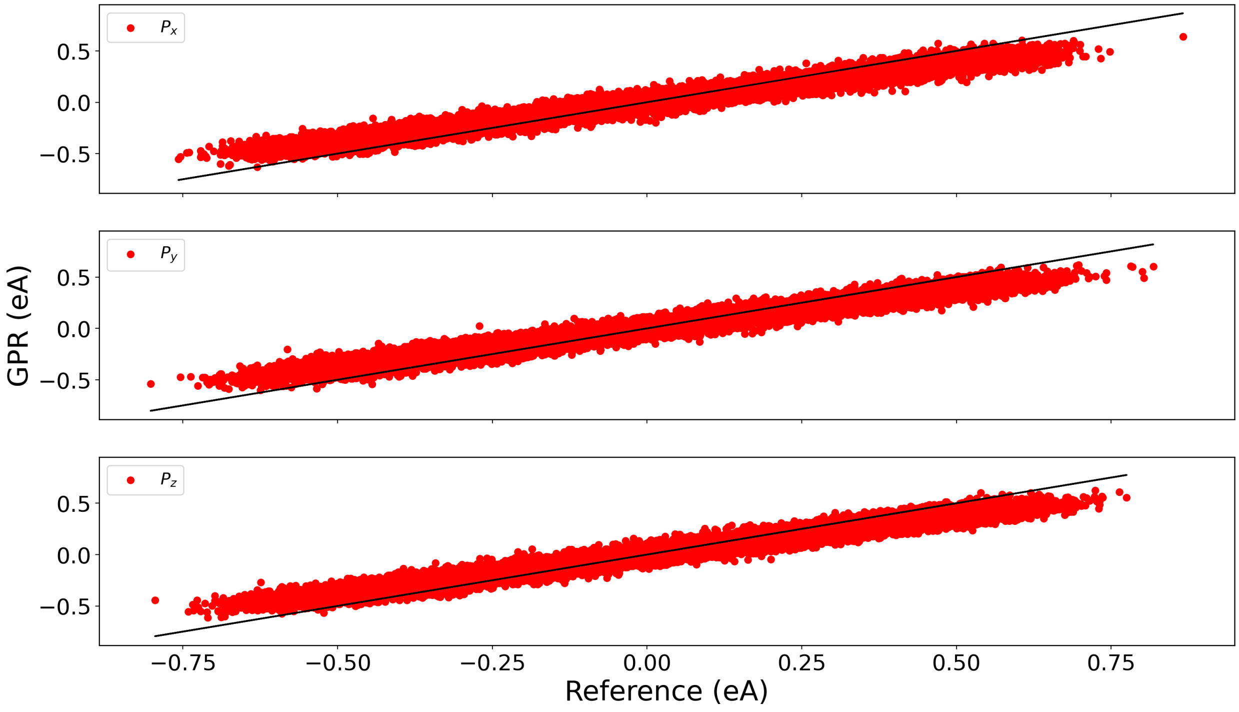

FIG. S11: Prediction of molecular P of ML-POLY-A model.

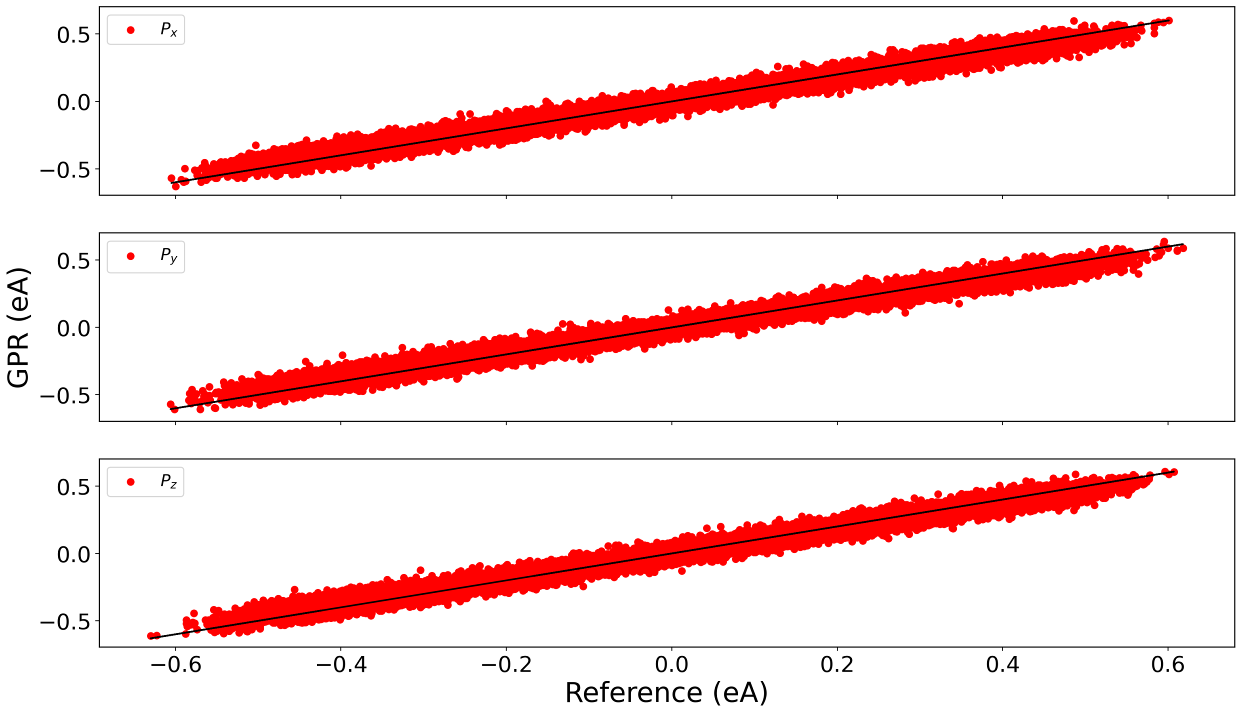

FIG. S12: Prediction of molecular P of ML-POLY-B model on water hexamers

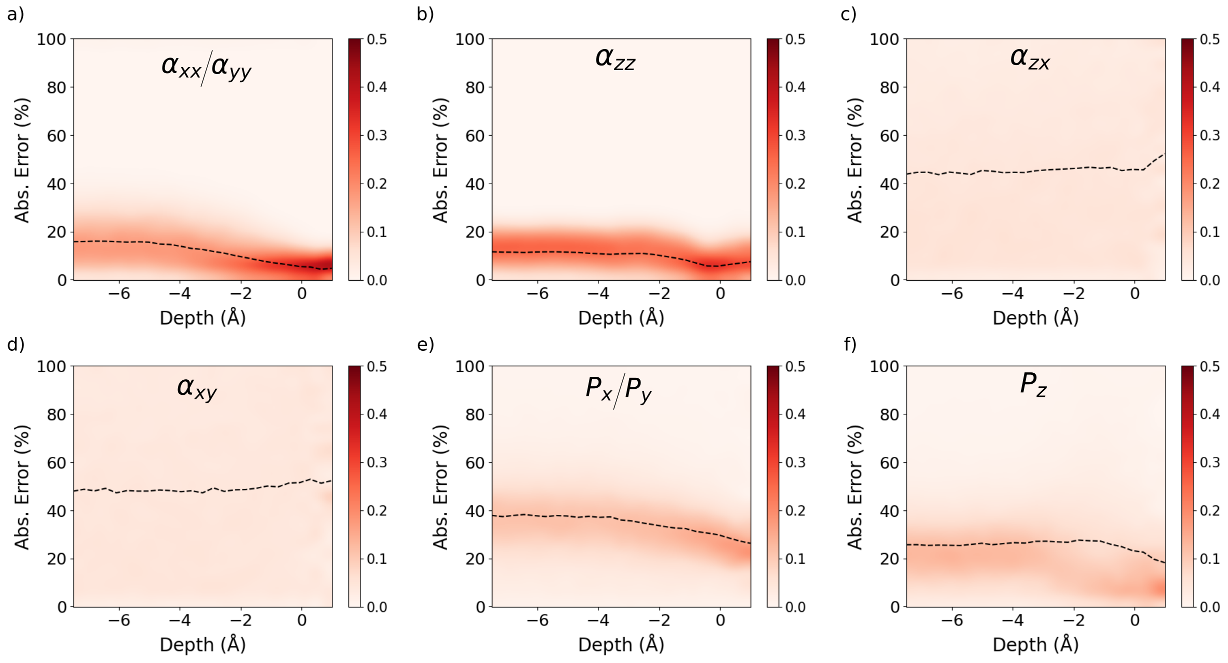

FIG. S13: Absolute error histograms for molecular α and P predictions as a function of distance to the interface using the ML-POLY-A model. Depth distances were computed with respect to the instantaneous interface[30]. Black dashed lines correspond to average values.

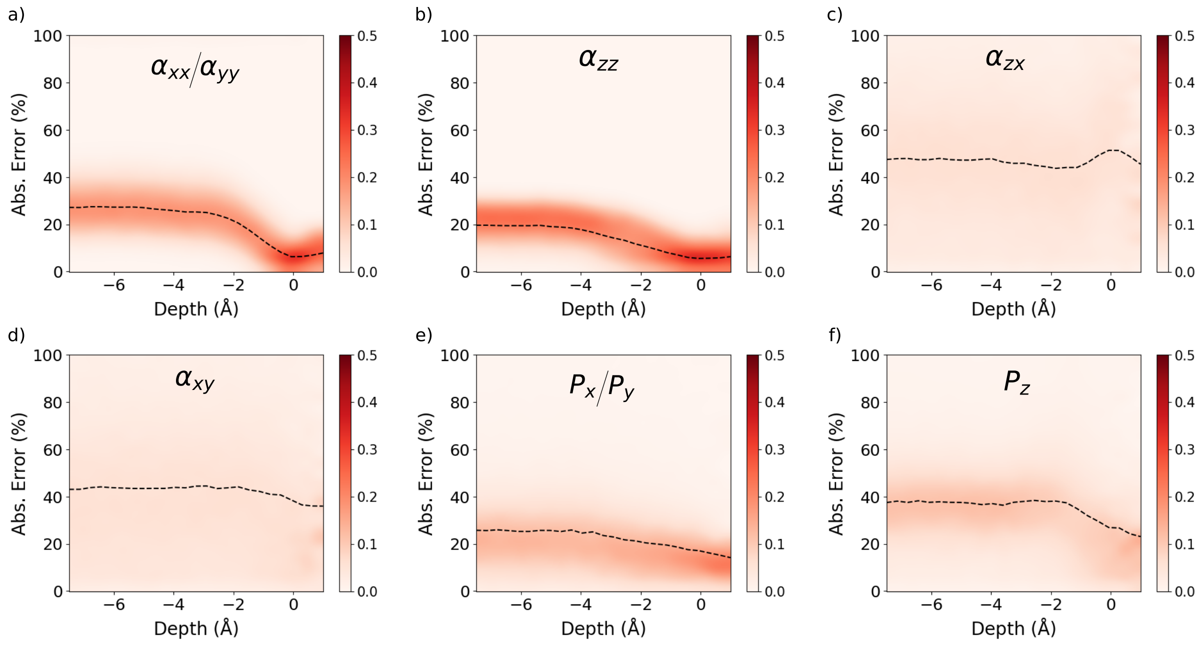

FIG. S14: Same as S13 for ML-POLY-B model.

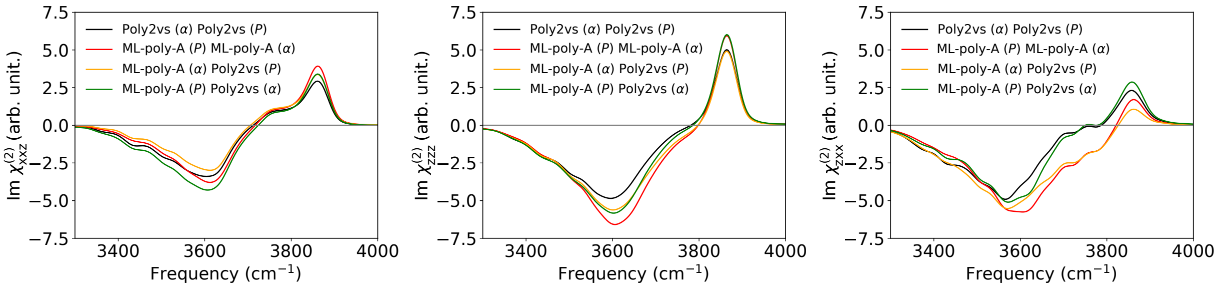

FIG. S15: Imaginary part of non-zero and independent $\chi^{(2)}$ components of the water/air interface using different combinations of POLY2VS and ML-POLY-A P and α surfaces for a slab geometry made of 160 water molecules. This test was performed for a shorter trajectory, thus the increased noise in the signal with respect other figures.

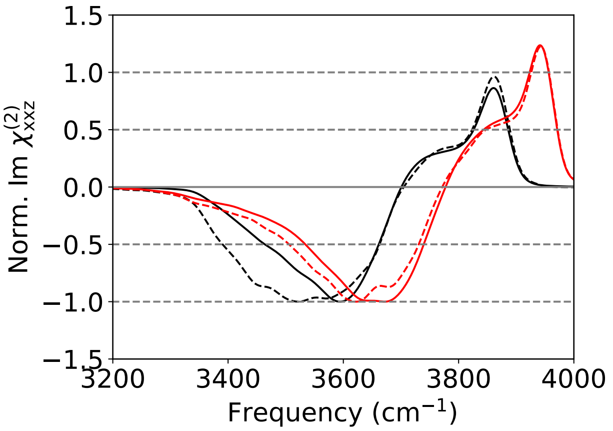

FIG. S16: Imaginary part of $\chi^{(2)}_{\mathrm{xxz}}$ of the water/air interface using the POLY2VS P and α surfaces combined with POLY2VS potential energy surface (PES) (black) and HDNNP trained on revPBE0-D3 data PES (red). Solid and dashed lines correspond to calculation 1 Å and 4 Å cutoff values, respectively. Data has been normalized at the H-bonded band to ease comparison.

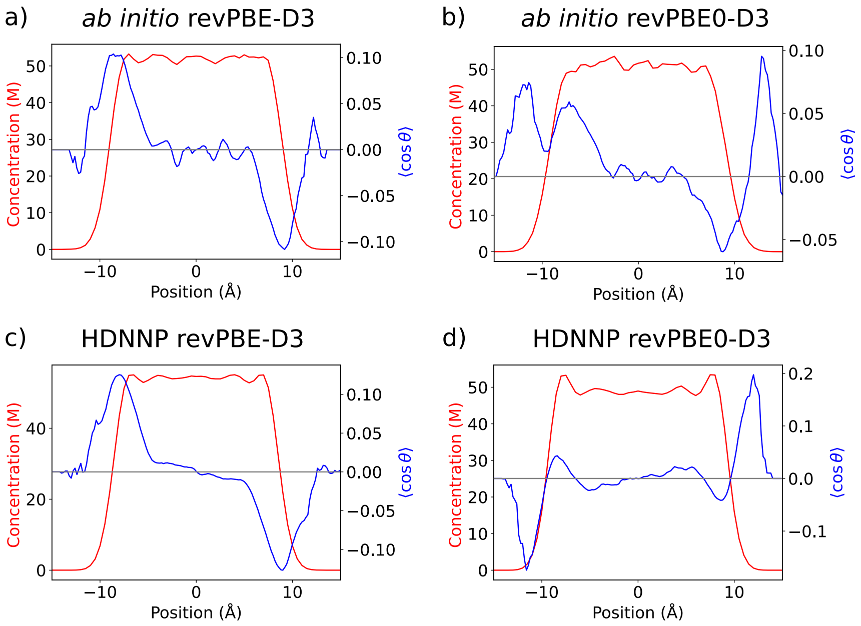

FIG. S17: Water concentration and cos(θ) profiles along the direction orthogonal to the slab surface. The center of the slab is set 0 Å. θ corresponds to the angle between the water bisector and the direction orthogonal to the water/air interface.

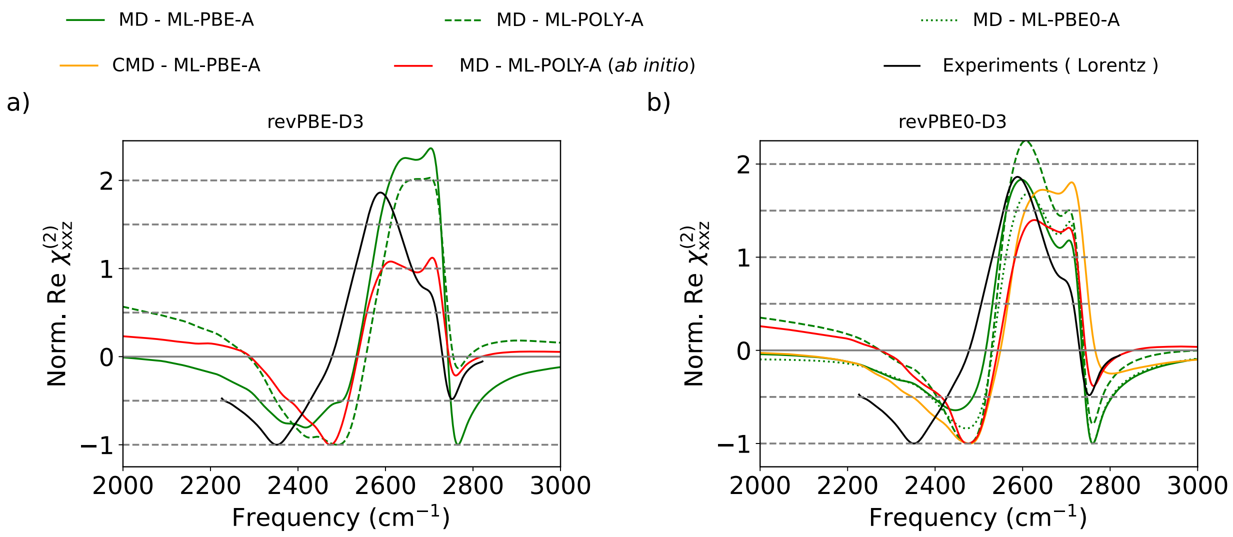

FIG. S18: Normalized Real part of χ_{xxz} spectra of the D_2O/air interface at 300K. Simulated spectra using revPBE-D3 (left) and revPBE0-D3 (right) exchange correlation (XC) functionals. Classical MD simulations using high-dimensional neural networks (HDNNP)s are presented by solid (ML-PBE-A), , dashed (ML-POLY-A), and dotted (ML-PBE0-A) green lines. centroid molecular dynamics (CMD) simulations are depicted with solid orange lines while results based on direct *ab initio* trajectories are depicted with red lines. Experimental spectra are depicted with solid black lines and horizontal gray lines have been added to guide the eye. Spectra have been rigidly shifted to match experimental spectra. Experimental spectra are corrected by the appropriate Fresnel factors, assuming the Lorentz model (black dotted lines) for the interfacial dielectric constant[31]. To allow a comparative analysis, we present the spectra, normalized such that the minimum intensity has an intensity of minus one.

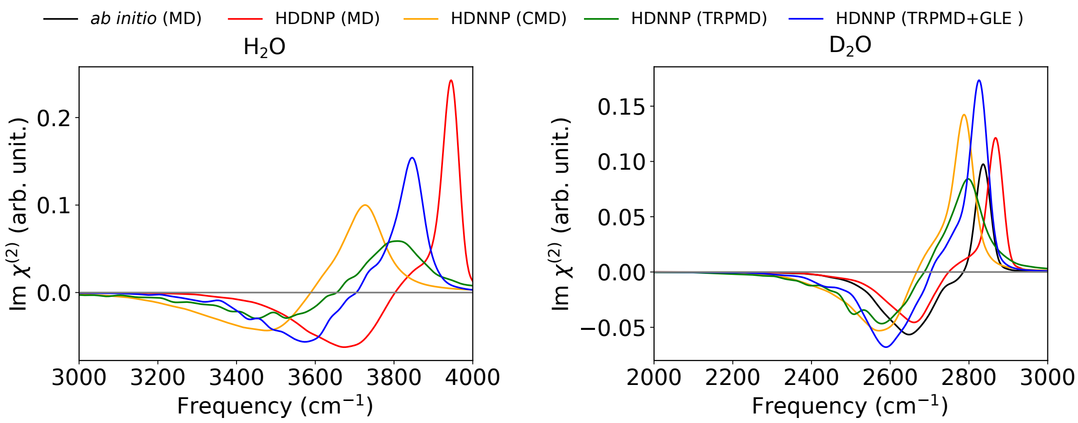

FIG. S19: Imaginary part $\chi^{(2)}$ of the water/air interface calculated using the ssVVCF approximations[32] and different molecular dynamics (MD, red line), centroid molecular dynamics (CMD, orange line), thermostated ring polymer molecular dynamics (TRPMD, orange line), and thermostated ring polymer molecular dynamics tuned with a generalized Langevin thermostat (TRPMD+GLE, blue line). All simulations were performed at 300K employing the HDNNP trained on revPBE0-D3 data.

REFERENCES

[1] V. Kapil, M. Rossi, O. Marsalek, R. Petraglia, Y. Litman, T. Spura, B. Cheng, A. Cuzzocrea, R. H. Meißner, D. M. Wilkins, B. A. Helfrecht, P. Juda, S. P. Bienvenue, W. Fang, J. Kessler, I. Poltavsky, S. Vandenbrande, J. Wieme, C. Corminboeuf, T. D. Kühne, D. E. Manolopoulos, T. E. Markland, J. O. Richardson, A. Tkatchenko, G. A. Tribello, V. V. Speybroeck, and M. Ceriotti, Comp. Phys. Commun. **236**, 214 (2019).

[2] A. P. Thompson, H. M. Aktulga, R. Berger, D. S. Bolintineanu, W. M. Brown, P. S. Crozier, P. J. i. t. Veld, A. Kohlmeyer, S. G. Moore, T. D. Nguyen, R. Shan, M. J. Stevens, J. Tranchida, C. Trott, and S. J. Plimpton, Comput. Phys. Commun. **271**, 108171 (2022).

[3] A. Singraber, J. Behler, and C. Dellago, J. Chem. Theory Comput. **15**, 1827 (2019).

[4] C. Schran, K. Brezina, and O. Marsalek, J. Chem. Phys. **153**, 104105 (2020).

[5] Y. Litman, K.-Y. Chiang, T. Seki, Y. Nagata, and M. Bonn, arXiv , 2210.01527 (2022).

[6] G. Bussi, D. Donadio, and M. Parrinello, J. Chem. Phys. **126**, 014101 (2007).

[7] O. Marsalek and T. E. Markland, J. Phys. Chem. Lett. **8**, 1545 (2017).

[8] T. Hasegawa and Y. Tanimura, J. Phys. Chem. B **115**, 5545 (2011).

[9] T. Ohto, M. Dodia, J. Xu, S. Imoto, F. Tang, F. Zysk, T. D. Kühne, Y. Shigeta, M. Bonn, X. Wu, and Y. Nagata, J. Phys. Chem. Lett. **10**, 4914 (2019).

[10] N. K. Kaliannan, A. H. Aristizabal, H. Wiebeler, F. Zysk, T. Ohto, Y. Nagata, and T. D. Kühne, Mol. Phys. **118**, 1620358 (2020).

[11] D. R. Moberg, S. C. Straight, and F. Paesani, J. Phys. Chem. B **122**, 4356 (2018).

[12] T. D. Kühne, M. Iannuzzi, M. Del Ben, V. V. Rybkin, P. Seewald, F. Stein, T. Laino, R. Z. Khaliullin, O. Schütt, F. Schiffmann, *et al.*, J. Chem. Phys. **152**, 194103 (2020).

[13] J. VandeVondele and J. Hutter, J. Chem. Phys. **127**, 114105 (2007).

[14] S. Goedecker, M. Teter, and J. Hutter, Phys. Rev. B **54**, 1703 (1996).

[15] C. Hartwigsen, S. Gœdecker, and J. Hutter, Phys. Rev. B **58**, 3641 (1998).

[16] M. Guidon, J. Hutter, and J. VandeVondele, J. Chem. Theory Comput. **6**, 2348 (2010).

[17] M. Guidon, J. Hutter, and J. VandeVondele, J. Chem. Theory Comput. **5**, 3010 (2009).

[18] R. King-Smith and D. Vanderbilt, Phys. Rev. B **47**, 1651 (1993).

[19] R. Resta, Rev. Mod. Phys. **66**, 899 (1994).

[20] A. Grisafi, D. M. Wilkins, G. Csányi, and M. Ceriotti, Phys. Rev. Lett. **120**, 036002 (2018).

[21] D. M. Wilkins, A. Grisafi, Y. Yang, K.-U. Lao, R. A. DiStasio, and M. Ceriotti, Proc. Natl. Acad. Sci. **116**, 3401 (2019).

[22] V. Kapil, D. M. Wilkins, J. Lan, and M. Ceriotti, J. Chem. Phys. **152**, 124194 (2020).

[23] A. Grisafi and M. Ceriotti, J. Chem. Phys. **151**, 204105 (2019).

[24] K. Inoue, Y. Litman, D. M. Wilkins, Y. Nagata, and M. Okuno, J. Phys. Chem. Lett. **14**, 3063 (2023).

[25] C. Schran, F. L. Thiemann, P. Rowe, E. A. Müller, O. Marsalek, and A. Michaelides, Proc. Nat. Ac. Sci. **118**, e2110077118 (2021).

[26] M. Rossi, M. Ceriotti, and D. E. Manolopoulos, J. Chem. Phys. **140**, 234116 (2014).

[27] M. Rossi, V. Kapil, and M. Ceriotti, J. Chem. Phys. **148**, 102301 (2018).

[28] S. Jang and G. A. Voth, J. Chem. Phys. **111**, 2371 (1999).

[29] J. Cao and G. A. Voth, J. Chem. Phys. **100**, 5106 (1994).

[30] A. P. Willard and D. Chandler, J. Phys. Chem. B **114**, 1954 (2010).

[31] X. Yu, K.-Y. Chiang, C.-C. Yu, M. Bonn, and Y. Nagata, J. Chem. Phys. **158**, 044701 (2023).

[32] T. Ohto, K. Usui, T. Hasegawa, M. Bonn, and Y. Nagata, J. Chem. Phys. **143**, 124702 (2015).
