# Supplementary material for: Fully First-Principles Surface Spectroscopy with Machine Learning
Source: J Phys Chem Lett. 2023 Sep 6;14(36):8175–82. doi: 10.1021/acs.jpclett.3c01989 (PMC10510433; doi:10.1021/acs.jpclett.3c01989)
Supplement: Supplementary file 2 — jz3c01989_si_002.pdf [file jz3c01989_si_002.pdf]

jz-2023-01989s.R1

Name: Peer Review Information for "Fully First-Principles Surface Spectroscopy with Machine Learning"

First Round of Reviewer Comments

Reviewer: 1

Comments to the Author

The authors presented a first-principles method to simulate SFG spectra of the air/water interface by combining a neural network potential for the potential energy surface and a Gaussian process regression for dipole moment/polarizability. The presented machine learning method is well validated and it is useful to simulate SFG from classical trajectories, I recommend the publication of the manuscript after addressing a few comments below.

・I wonder how the authors performed ML-PBE0-A simulation while the RMSE of the polarizability of this model is absent in Table 1.

・The prediction plots are provided only for ML-POLY (Figs S4-S9). These plots for ML-PBE should be given as well.

・I wonder the statement in page 2, "This approximation cannot predict the spectral differences observed with different combinations of laser polarization and it is unable to capture important spectral features originating from vibrational coupling."

I think that, with velocity-velocity correlation function formalism, it is possible to simulate the different combinations of laser polarization and include vibrational couplings (cross-correlation).

・"By comparing the CMD and TRPMD results, we deduce that the curvature problem is responsible for an additional 10 cm−1 red-shift."

Did the authors compare the CMD and TRPMD in fig. S16? If so, what is the implication of the TRPMD + generalized Langevin thermostat simulation?

Reviewer: 2

Comments to the Author

The paper deals with a machine-learning approach for sum-frequency generation (SFG) spectra.

I have the following comments:

- What influence does it have that only 1000 frames were used for ML-PBE0-A and ML-POLY-A

but much more for ML-POLY-B?

- The authors should mention about the electric dipole approximation used.
- The authors should include a discussion (also reviews etc.) about previous machine learning approaches, especially for electric dipole/polarisability predictions in this context.
- How deal the authors with Fresnel factors?
- It is not clear why Eq. 4 has the proposed form.
- What is the reason chi_xxx is predicted worse (p. 4)?
- What can be done to cure the artifacts regarding the intensity ratio of free and HB O-D vibrat ions?
- More detailed comparison of computational costs would be desirable.

For deeper understanding, it would be good to have a more detailed description of the machine learning approaches used in the main text.

The various machine learning models used for the different ingredients needed for sum-frequency generation spectra have already been published in other works, therefore I consider this work to be of limited novelty.

Moreover, only water/air interfaces have been analysed although there are many other systems used for SFG (also e.g. solid state-air/water interfaces).

Author's Response to Peer Review Comments:

Dr Yair Litman
Walter Benjamin Research Fellow

Yusuf Hamied Department of Chemistry
Lensfield Rd, Cambridge CB2 1EW
Tel: +44-01223 336340
Email: yl899@cam.ac.uk

Aug 25, 2023

Dear Prof. Editor
J. Phys. Chem. Lett.

We were happy to read the positive assessment of our paper by one of the reviewers. We have carefully revised the manuscript to address all major concerns of the reviewers and we have also addressed all editorial requests. Point-by-point answers are enclosed below.

All the other reviewers' concerns could be addressed by clarifying some sentences in the main text, extending the discussion on specific points, and including numerical tests and information in the supplemental information. We have considerably extended the description of Eq. 4 which is central to the proposed approach, included a brief discussion of other machine learning approaches relevant in the present context, and clarified why the current work is the only machine learning approach proven to be suitable to simulate surface-specific spectroscopy. Additionally, a clarification regarding the different methods employed to approximate quantum dynamics, the main approximations used to derive the equation used to simulate the sum-frequency generation spectra, Eq 1, and a detailed evaluation of the computational cost have been incorporated into the manuscript.

We hope that the current version of the manuscript meets the requirements for publication in J. Chem. Phys. Letter.

Dr. Yair Litman and Dr. David M. Wilkins, on behalf of all co-authors

Formatting Notes from the Editorial Office:

1. One or more of your figures and tables includes a reference citation. Please confirm that this pertains only to data and not the figure itself. If it pertains to the use of a published image, permissions must be secured for any graphics NOT originally published by ACS or for Open Access content. Permission is needed if you are using another publisher's or copyright owner's figures/tables verbatim, adapting/modifying them, or using them in part. If the images are from an Open Access publisher, please confirm.

AUTHORS: We confirm that it pertains only to the data and not the figure itself.

2. In both the main file and the supporting information, fix the style of all references to use JPCL formatting (check all references carefully). ***JPC Letters reference formatting requires that journal references should contain: () around numbers; author names; article title (titles entirely in title case or entirely in lower case); abbreviated journal title (italicized); year (bolded); volume (italicized); and pages (first-last). Book references should contain author names; book title (in the same pattern); publisher; city; and year. Websites must include date of access.

3. There is a discrepancy between an author name in the manuscript/Paragon Plus (Yair Litman) and the name on the SI file (Y. Litman). Please correct the name so that it is the same in all locations.

4. Please add the corresponding author's email address to the title page of the SI file.

5. Please number SI pages in the following format: “S1, S2…”

6. Please label all SI graphics in the following format: “Figure S1, S2…”, “Scheme S1, S2….” or “Table S1, S2…”, etc.

7. Please include annotated version(s) of your revised publication file(s) with colored text or highlights indicating the revisions that you have made, and upload them as “Supporting Information for Review Only." Please also upload “clean” copies for publication. (No highlighting, annotations, or colored text.)

AUTHORS: The points 2-7 have been addressed as requested.

Reviewer(s)' Comments to Author:

Reviewer: 1

Recommendation: This paper is publishable subject to minor revisions noted. Further review is not needed.

Comments:
The authors presented a first-principles method to simulate SFG spectra of the air/water interface by combining a neural network potential for the potential energy surface and a Gaussian process regression for dipole moment/polarizability. The presented machine learning method is well validated and it is useful to simulate SFG from classical trajectories, I recommend the publication of the manuscript after addressing a few comments below.

REVIEWER: I wonder how the authors performed ML-PBE0-A simulation while the RMSE of the polarizability of this model is absent in Table 1.

AUTHORS: We thank the reviewer for noticing this point. The ML-PBE0-A model was trained on PBE0 data for the polarization. Due to the computational cost associated with the polarizability calculations, the polarizability part of the ML-PBE0-A model was kept at the PBE level. As noted by Shang and co-workers (New J. Phys. 20, 073040 (2018)), the changes in polarization with atomic displacement are surprisingly similar between PBE and PBE0. This observation justifies our choice of focusing exclusively on the effect of the changing electronic structure description of the polarization.

The following clarification has been added to the text:

Page 12:*"… we also tested the ML-PBE0-A model, in which the polarization was fitted to PBE0 reference data but the polarizability was kept at the PBE level."*

REVIEWER: The prediction plots are provided only for ML-POLY (Figs S4-S9). These plots for ML-PBE should be given as well.

AUTHORS: We thank the reviewer for pointing out this omission. We have included the correlation plots for the ML-PBE-A and ML-PBE0-A models in Figures S8-S10 in the supporting information.

We present the figures below for the reviewer's convenience

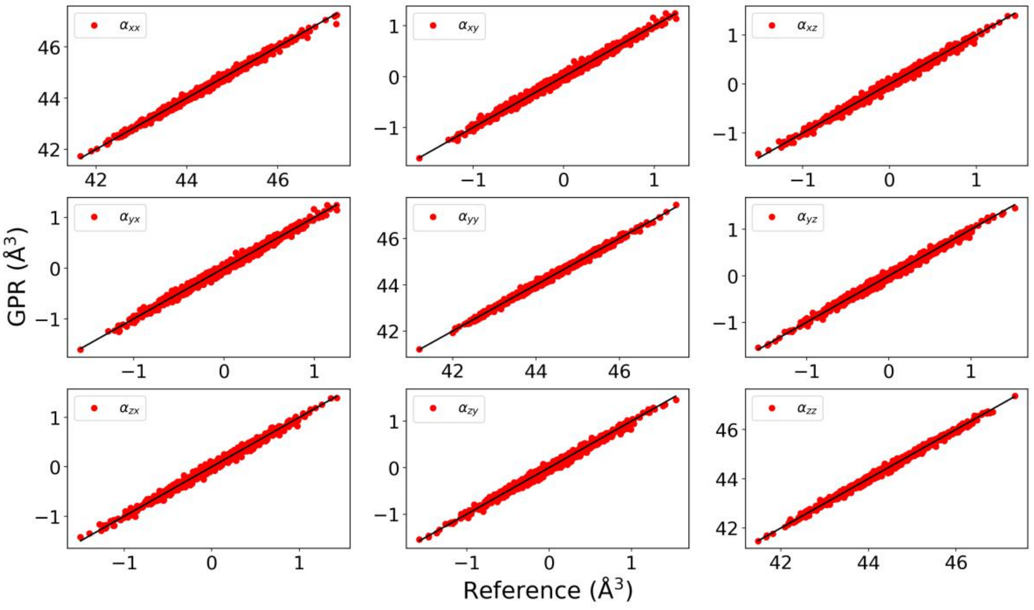

Figure 1: Prediction of total α components for ML-PBE-A model.

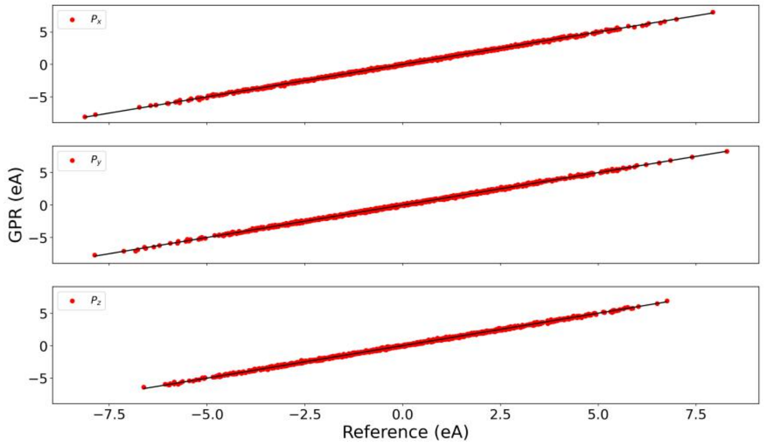

Figure 2: Prediction of total P for ML-PBE-A model.

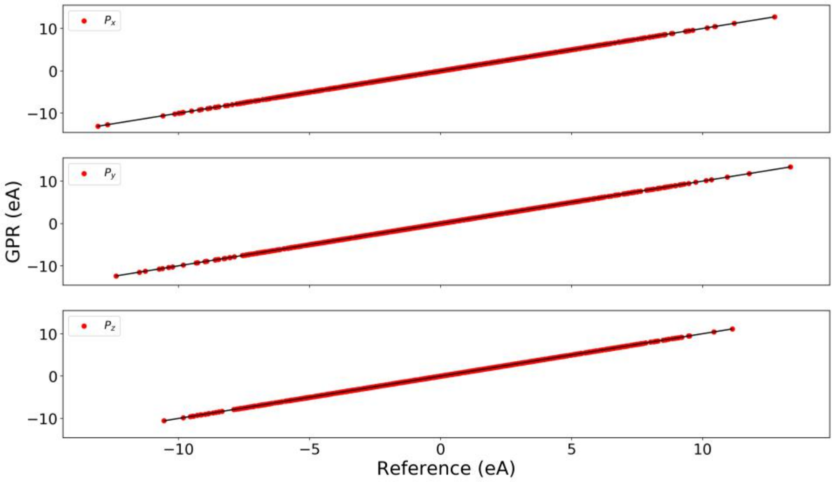

Figure 3: Prediction of total P for ML-PBE0-A model.

REVIEWER: I wonder the statement in page 2, "This approximation cannot predict the spectral differences observed with different combinations of laser polarization and it is unable to capture important spectral features originating from vibrational coupling." I think that, with velocity-velocity correlation function formalism, it is possible to simulate the different combinations of laser polarization and include vibrational couplings (cross-correlation).

AUTHORS: We thank the reviewer for this important question. The surface velocity-velocity correlation function formalism (SVVCF) approximates the diagonal elements of the transition polarizability moment matrix by i) taking an isotropic average of the diagonal elements and ii) and neglecting the off-diagonal ones (J. Chem. Phys. 143, 124702 (2015)). Mathematically, this can be expressed as i) $\partial\alpha_{xx}/\partial r = \partial\alpha_{yy}/\partial r = \partial\alpha_{zz}/\partial r$, and ii) $\partial\alpha_{xy}/\partial r = \partial\alpha_{xz}/\partial r = \partial\alpha_{yz}/\partial r = 0$, where α_{ab} is the *ab* component of the molecular polarizability, and *r* represents the O-H distance. According to this approximation, and contrary to what it is observed in experiments, the VSFG spectra measured with ssp and ppp polarization are expected to be identical since they are related to α_{yy} and α_{zz} elements, respectively. Moreover, the spectra obtained with ppp polarization, which is related to α_{xz} elements, cannot be described at all within the SVVCF formalism.

We have clarified the corresponding sentence on page 4 which now reads:

Page 4: "*This approximation cannot predict the spectral differences observed with different combinations of laser polarization, since due to the approximations involved, it (incorrectly) predicts that $\chi^{(2)}_{xxz} = \chi^{(2)}_{zzz}$ and $\chi^{(2)}_{zxz} = 0$. Furthermore, it is unable to capture important spectral features originating from vibrational coupling.*[16–18]"

REVIEWER: "By comparing the CMD and TRPMD results, we deduce that the curvature problem is responsible for an additional 10 cm−1 red-shift." Did the authors compare the CMD and TRPMD in fig. S16? If so, what is the implication of the TRPMD + generalized Langevin thermostat simulation?

AUTHORS: We have compared CMD with TRPMD and TRPMD + generalized Langevin thermostat (TRPMD + GLE). The latter method uses specifically tailored thermostats based on the generalized Langevin equation to reduce the artificial broadening of the high-frequency peaks observed in the original TRPMD formulation.

We have added the following clarification to the main text:

Page 12: "We also compared with the latest refinement of the TRPMD method that employs thermostats based on the generalized Langevin equation that has been specifically designed to mitigate some of the artefacts associated with the original TRPMD formulation[55], arriving to the same conclusion (see Fig. S19 in the SI)."

Additional Questions:
Urgency: High

Significance: High

Novelty: Top 10%

Scholarly Presentation: High

Is the paper likely to interest a substantial number of physical chemists, not just specialists working in the authors' area of research?: Yes

Reviewer: 2

Recommendation: Reconsider as an article in The Journal of Physical Chemistry A/B/C.

Comments:
The paper deals with a machine-learning approach for sum-frequency generation (SFG) spectra.

I have the following comments:

REVIEWER: What influence does it have that only 1000 frames were used for ML-PBE0-A and ML-POLY-A but much more for ML-POLY-B?

AUTHORS: We thank the reviewer for raising this concern. The ML-XXX-A models were trained on bulk geometries with 32 water molecules per frame, while the ML-XXX-B models were trained on clusters up to hexamers. Ten thousand hexamers frames and one thousand bulk frames comprise 60 ,000 and 32,000 water molecules, respectively. Thus, both models have been trained on a comparable number of atomic environments. Moreover, while the training curves in the SI suggest that more training data will in principle decrease the training errors, the fact that both models show very similar performance (Fig. 1) suggests that additional data would not improve the accuracy of the predictions.

REVIEWER: The authors should mention about the electric dipole approximation used.

AUTHORS: We agree with the reviewer that the limitation of Eq. 1 should be explicitly stated. We have added a phrase in this regard and made explicit the absence of quadrupole contributions.

Page 14: "*We note that the modelling of the VSFG spectra described by Eq. 1 is derived in the electric dipole approximation.[5] We believe that applying the current methodology to a more accurate reference data set would finally resolve existing controversies related to, for example, the relevance of quadrupole contributions in the water bending mode.[65,66]*"

REVIEWER: The authors should include a discussion (also reviews etc.) about previous machine learning approaches, especially for electric dipole/polarisability predictions in this context.

AUTHORS: We agree with the reviewer on this point. We have included a paragraph in this regard in the last part of the manuscript.

Page 14: "Several other ML approaches with varying architectures have been reported in the five years to predict molecular dipole moments,[58–61] and linear vibrational spectroscopy.[62–64] However, attempts to simulate VSFG spectroscopy have been lagging behind, most likely due to the destructive interference of the signal, which made those approaches inappropriate for this target. While the strategy presented here could be easily applied to other kernel regression models that use local representations, it remains to be seen if similar ideas could be implemented on neural network architectures."

REVIEWER: How deal the authors with Fresnel factors?

AUTHORS: The experimental data presented in Fig. 2 and Fig. 3 has been corrected with the corresponding Fresnel factors assuming the Lorentz model for the interfacial dielectric constant (see caption of Fig. 2 and Fig. 3). As discussed in J. Chem. Phys. 158, 044701 (2023), the Lorentz model predicts Fresnel factor corrections that are in excellent agreement with more sophisticated models that incorporate the detailed inhomogeneous interfacial dielectric profile. For this reason, a direct comparison with the simulated spectra computed using Eq. 1 can be made without further corrections.

REVIEWER: It is not clear why Eq. 4 has the proposed form.

AUTHORS: We thank again the reviewer pointing out that the justification of Eq. 4 could be improved.

As discussed on Page 6, “The calculation of $\chi^{(2)}$ for a slab geometry needs to be done with care since the contributions of opposite interfaces interfere destructively and lead to a vanishing response.” Eq. 4 solves this problem by:

i) flipping the sign of the molecular contributions to the polarization that emerge from water molecules that are in the vicinity of the bottom interface (first condition in Eq. 4).
ii) setting to zero the contributions to the polarization corresponding to water molecules that are in the vicinity of the slab centre (second condition in Eq. 4)
iii) adding a smooth transition between i and ii (third condition in Eq. 4)

We note that in reviewing this point, we found a small omission inside the sign function in Eq. 4 that has been corrected in the current version. We have re-expressed Eq. 4 in a, mathematically equivalent way that we believe it might be clearer for the reader.

We have also added the following clarification below Eq. 4

Page 7: *“The first condition in Eq. 4 flips the sign of the molecular contributions to the polarization that emerge from water molecules that are in the vicinity of the bottom interface, the second condition sets to zero the contributions to the polarization corresponding to water molecules that are in the vicinity of the slab centre, and the third condition is a smooth transition between the previous two conditions. In this way, the signal of the opposite interfaces interfere constructively, leading to a non-vanishing VSFG signal”*

REVIEWER: What is the reason chi_xxx is predicted worse (p. 4)?

AUTHORS: $\chi^{(2)}_{xxx}$ is zero due to due to the factor that the current x-y is isotropic and the symmetry of the polarizability tensor. The $\chi^{(2)}_{zxx}$ is the component that is described least accurately.

This point is acknowledged in the text and discussed on page 14:

“We showed that the off-diagonal components of the α tensor for water are more difficult to learn due to their smaller magnitude when compared to the diagonal ones, which leads to a poorer description of certain $\chi^{(2)}$ matrix elements. Since spherical components mix diagonal and off-diagonal elements, it is not possible to train the off-diagonal elements exclusively with current SA-GPR implementations.”

REVIEWER: What can be done to cure the artifacts regarding the intensity ratio of free and HB O-D vibrat ions?

AUTHORS: We thank the reviewer for asking this important question. The quality of the final VSFG spectra depends on the quality of the potential energy, polarization and polarizability surfaces. These in turn depend on the quality of the reference data and the possible further inaccuracies introduced by the associated machine learning model. Since we show that the later contribution is comparatively small, in particular for $\chi^{(2)}_{xxz}$, we are confident that the main source of inaccuracy is the quality of the reference data.

This issue is discussed manuscript in two opportunities:

Page 11 and 12: (slightly reworded in the current version) *“However, for all tested cases, irrespective of the PES, the theoretical predictions overestimate the intensity ratio between the free O-D and the hydrogen-bonded band. This underestimation has been reported by Paesani and co-workers when induction effects arising from interactions between individual molecules are neglected.[47] Since SA-GPR can capture local induction effects, the large discrepancy is attributed to the quality of the underlying reference data.”*

Page 14: *“Since the discrepancies between the theoretical and the experimental spectra are larger than the training error of the SA-GPR models, we attribute the largest source of error to the reference data used to describe the P and α surfaces. A further study using electronic structure methods beyond DFT to obtain more accurate P and α surfaces is urgently needed”*

REVIEWER: More detailed comparison of computational costs would be desirable.

AUTHORS: We thanks the reviewer for this request and have now provided a more detail analysis of the computational cost in the supporting information.

Page S6: "*The computational cost associated with the simulation of the VSFG spectra using the presented machine learning (ML) approach can be divided into four parts: i) Obtaining the reference data ($C_{ref\text{-}data}$), ii) Training the ML model (C_{train}), iii) Performing the molecular dynamics simulations (C_{MD}), and iv) Evaluation of the polarization and polarizability ($C_{P,\alpha}$). The cost of point ii) is negligible when compared to the other points so it is not considered further in this analysis. Furthermore, point i) can be obtained from a single 20 ps long molecular dynamics trajectory*[25]*, which is 1000 times shorter than the trajectory length required to compute a VSFG spectrum. If one is interested in computing just a single VSFG spectra, $C_{ref\text{-}data}$ becomes the determining factor of the total cost. This represents a saving of three orders of magnitude when compared to direct ab initio simulations. On the contrary, if one is interested in obtaining spectra at different conditions, such as at slightly different temperatures, various isotopic mixtures, or using different methods to include nuclear quantum effects NQEs, then C_{MD} and $C_{P,\alpha}$ become the bottleneck. However, with the current implementations, the computational cost of the former contribution is 100-200 times smaller. Finally, the evaluation of the polarization and polarizability for a single configuration using the SA-GPR model and direct ab initio calculations have a computational cost of 0.01 CPU hours and 1000 CPU hours (Intel® Xeon® E5-2690 v3 @ 2.60GHz), respectively. In this scenario, the computational saving goes up to a factor of 10^5.*"

REVIEWER: For deeper understanding, it would be good to have a more detailed description of the machine learning approaches used in the main text. The various machine learning models used for the different ingredients needed for sum-frequency generation spectra have already been published in other works, therefore I consider this work to be of limited novelty. Moreover, only water/air interfaces have been analysed although there are many other systems used for SFG (also e.g. solid state-air/water interfaces).

AUTHORS: We respectfully disagree with the Reviewer's view on the limited novelty of the current manuscript. As stated in our introduction, and in the previous answers, current approaches to simulate VSFG spectroscopy cannot capture the spectral differences observed with different combinations of laser polarization or cannot handle bond breaking or formation. While we agree with the reviewer that the different machine learning techniques used in this work were reported several years ago, and have been used to compute simple vibrational spectroscopies such as Raman and IR, VSFG is a much more challenging type of spectroscopy due to the previously discussed signal cancellation between opposite interfaces and the need to simulate much longer trajectories to reach convergence. Indeed, while fully *ab initio* Raman and IR spectroscopies have been reported, see for example J. Phys. Chem. Lett. **8**, 1545–155 (2017)), a fully first principles simulation of the VSFG spectra of the water/air interface, arguably the most studied interface, has been lacking in literature. The current work not only presents a simple and elegant way to obtain VSFG spectra at *ab initio* level and with a massive computation saving, but also clearly exposes serious inaccuracies of several commonly used exchange-correlation functionals, and shows the limitations of current approximation to describe nuclear quantum dynamics. Furthermore, we were able to identify the main source of inaccuracies and to propose a clear pathway towards which future efforts should be focused. Finally, the present approach is general since it does not make use of specific information on the nature of the system, such as physical constraints designed for water or the availability of a surrogate model. Therefore, we believe that it would be a fundamental stepping stone to study reactivity in aqueous complex interfaces that have remained inaccessible until now.

Additional Questions:
Urgency: Moderate

Significance: High

Novelty: Moderate

Scholarly Presentation: High

Is the paper likely to interest a substantial number of physical chemists, not just specialists working in the authors' area of research?: No
